# Structured Light Projection Using Image Guide Fibers for In Situ Photo‐biofabrication

**DOI:** 10.1002/adma.202419350

**Published:** 2025-04-29

**Authors:** Parth Chansoria, Michael Winkelbauer, Shipin Zhang, Jakub Janiak, Hao Liu, Dimitar Boev, Andrea Morandi, Rachel Grange, Marcy Zenobi‐Wong

**Affiliations:** ^1^ Department of Health Sciences and Technology Institute for Biomechanics Tissue Engineering and Biofabrication Group ETH Zürich 8093 Switzerland; ^2^ Department of Physics Institute for Quantum Electronics Optical Nanomaterial Group ETH Zürich 8093 Switzerland

**Keywords:** biofabrication, collagen, gelatin, image guide fiber, in situ, multiwavelength

## Abstract

Light‐based biofabrication techniques have revolutionized the field of tissue engineering and regenerative medicine. Specifically, the projection of structured light, where the spatial distribution of light is controlled at both macro and microscale, has enabled precise fabrication of complex three dimensional structures with high resolution and speed. However, despite tremendous progress, biofabrication processes are mostly limited to benchtop devices which limit the flexibility in terms of where the fabrication can occur. Here, a Fiber‐assisted Structured Light (FaSt‐Light) projection apparatus for rapid in situ crosslinking of photoresins is demonstrated. This approach uses image‐guide fiber bundles which can project bespoke images at multiple wavelengths, enabling flexibility and spatial control of different photoinitiation systems and crosslinking chemistries and also the location of fabrication. Coupling of different sizes of fibers and different lenses attached to the fibers to project small (several mm) or large (several cm) images for material crosslinking is demonstrated. FaSt‐Light allows control over the cross‐section of the crosslinked resins and enables the introduction of microfilaments which can further guide cellular infiltration, differentiation, and anisotropic matrix production. The proposed approach can lead to a new range of in situ biofabrication techniques which improve the translational potential of photofabricated tissues and grafts.

## Graphical abstract

Light‐based biofabrication is typically performed with single wavelength light sources and within benchtop devices. This work demonstrates FaSt‐Light (Fiber‐assisted Structured Light) as a new approach to achieve multiwavelength image projection using flexible image guide fibers, which enables a variety of applications for in situ biofabrication.

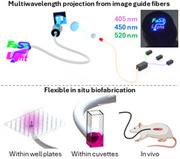

## 1 Introduction

Past two decades have seen a tremendous growth in the field of light‐based biofabrication.^[^ ^1^, ^2^ ^]^ A variety of processes have emerged to crosslink biomaterials,^[^ ^3^ ^]^ or to change functional properties of the materials across spatial domains.^[^ ^1^ ^]^ These processes offer high precision (up to 150 nm with two‐photon polymerization [2PP)),^[^ ^4^ ^]^ speeds (printing in several seconds using volumetric bioprinting),^[^ ^5^, ^6^ ^]^ and scalability (complex constructs spanning several centimeters can be printed using digital light projection [DLP)).^[^ ^7^ ^]^ These different approaches have found applications in the fabrication of acellular grafts for tissue repair,^[^ ^8^, ^9^ ^]^ or for biofabrication using living cells to engineer a variety of tissues such as cartilage,^[^ ^10^, ^11^ ^]^ liver,^[^ ^12^, ^13^ ^]^ cardiac^[^ ^14^, ^15^, ^16^ ^]^ and skeletal muscle,^[^ ^6^, ^17^ ^]^ etc. Spatially controlled light projections have also been used for inducing functional changes in photo‐responsive materials, such as spatially tuned presentation of growth factors or molecules for cell differentiation.^[^ ^18^, ^19^, ^20^ ^]^

While the light‐based techniques highlighted above can be used to fabricate complex constructs for a variety of regenerative applications, these approaches have been mostly limited to benchtop devices.^[^ ^21^, ^22^, ^23^ ^]^ The constructs are fabricated over a substrate or within a printing vial, and later post‐treated (washing, secondary crosslinking, additional sterilization, etc.), and cultured (for constructs with cells) before being used for implantation.^[^ ^21^, ^22^ ^]^ A benchtop fabrication approach limits the region and the containers these materials could be printed into. Furthermore, for applications where the constructs are being used for implantation, handling and surgical implantation of the grafts is often complicated. Notably, several studies have demonstrated in situ photo‐crosslinking of hydrogels for applications including diabetic wound healing,^[^ ^24^, ^25^ ^]^ treatment of myocardial infarction,^[^ ^26^, ^27^ ^]^ or corneal regeneration.^[^ ^28^, ^29^ ^]^ However, these approaches have crosslinked bulk resins around the tissue by projecting homogeneous beams through fibers or handheld light sources. Here, controlling the micro‐ and macro‐architecture of the in situ crosslinkable photoresins (usually hydrogels for biofabrication applications) can allow better cellular infiltration, growth, and tissue formation. To print complex structures, approaches such as tomographic printing would be complicated for in situ printing as it requires light projection from multiple orientations.^[^ ^22^ ^]^ In contrast, 2PP or DLP techniques have been used, since the light is projected from one direction.^[^ ^30^, ^31^, ^32^ ^]^ For instance, Urciolo and colleagues demonstrated 2PP in vivo bioprinting using a near infrared (NIR) laser (850 nm), which could penetrate up to several millimeters under the skin.^[^ ^32^ ^]^ In this study, a laser beam was moved to trace the outline of the constructs layer‐by‐layer, to create arbitrary shapes subcutaneously. In a similar study, Chen and colleagues demonstrated subcutaneous printing of photoresins by projecting images at NIR wavelength (980 nm),^[^ ^30^ ^]^ where crosslinking was achieved using upconversion nanoparticles coated with the photoinitiator lithium phenyl‐2,4,6‐trimethylbenzoylphosphinate (LAP). In contrast to these approaches, we hypothesized that coupling the projection apparatus to image guide fiber bundles would provide increased flexibility in directing light to otherwise inaccessible regions (e.g., tissues inside the body or in different types of resin containers, etc.). Image guide fiber bundles, also called coherent image guides or fiber optic imaging bundles, are stacked bundles of optical fibers which can convey optical spatial properties from one end of the fiber to another.^[^ ^33^, ^34^ ^]^ These fibers have been extensively used for imaging in regions where the approach area is small and difficult to reach (e.g., endoscopy).^[^ ^35^, ^36^ ^]^ The captured image is guided through the bundles into a camera sensor, which can then determine the depth and color of the images and render it on a user‐interface.^[^ ^33^, ^34^, ^37^ ^]^ Interestingly, reversing the function of the image guide fibers, where custom images generated from a projection apparatus are guided through the fibers for the crosslinking of materials, has not yet been explored for biofabrication applications. Notably, such a system is different from recent approaches using an optical fiber which emits a uniform light beam, and where the fiber needs to be mechanistically traversed to create complex crosslinked constructs.^[^ ^38^ ^]^

**Figure 1:**
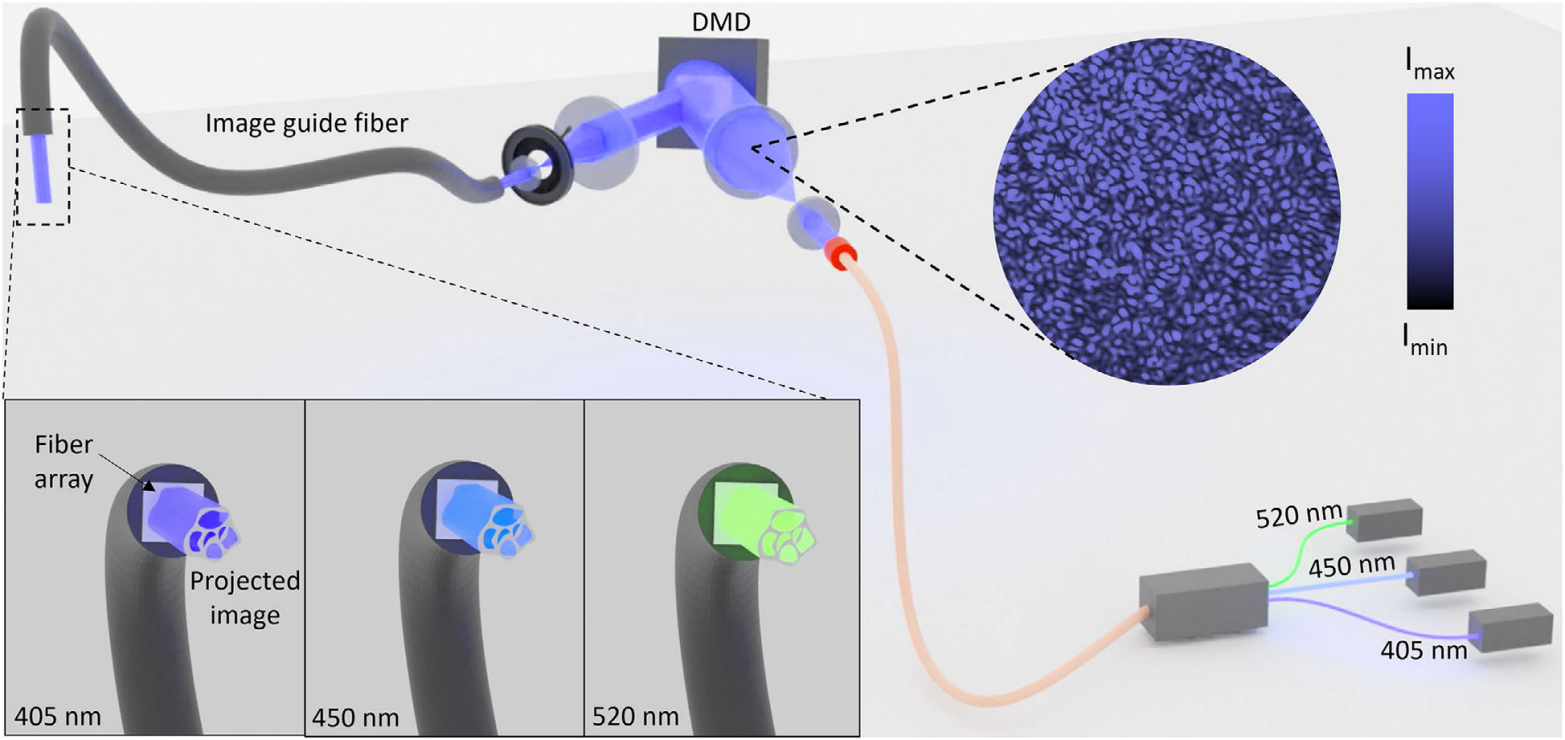
Schematic of the FaSt‐Light apparatus capable of projecting bespoke image patterns of different wavelengths through an image guide fiber bundle. Note that the contrast ratio of the speckle patterns (intrinsic to the laser light engine) has been increased in the illustration to demonstrate the effect of light filamentation due to optical modulation instability.

In this work, we demonstrate a **F**iber‐**a**ssisted **St**ructured **Light** (**FaSt‐Light**) projection apparatus (**Figure** **1**) for rapid in situ photo‐biofabrication using image guide fibers. The aspect of structured light entails two scales of control over the spatial distribution of light: (1) Macroscale structures (>50 µm) imparted through the digital micromirror device (DMD) in the projection system which controls the image at multiple wavelengths projected through the fibers, allowing control over the cross‐section of the crosslinked resin, and (2) microscale control, which refers to the microfilamentation of light beams due to the optical modulation instability when the laser speckles interact with the photoresin, allowing the introduction of cell‐guiding microfilaments (2–8 µm) within the crosslinked resin. We have previously leveraged the combined effect of macro‐ and microscale structures for the fabrication of microfilamented scaffolds using filamented light (FLight) biofabrication, which is a benchtop approach to generate highly aligned tissue constructs.^[^ ^10^, ^17^ ^]^ In this work, we demonstrate a new system, which generates speckled images at multiple light wavelengths (405, 450, 520 nm), which are projected through image guide fiber bundles onto photoresins contained within in a variety of enclosures (cuvettes, well plates, or defects on the tissues). We investigate the resins crosslinked using the FaSt‐Light approach in terms of the resulting macro and microscale features and further elucidate the effects of fiber cross‐section area, projection distance, and lenses coupled to the fibers on the structures. Through in vitro experiments, we show that the FaSt‐Light apparatus can be used to biofabricate viable tissue constructs or grafts which promote cell‐guidance and infiltration. Furthermore, we also demonstrate selected proof‐of‐concept applications where the approach could be used for the crosslinking of structured photoresins in vivo, which can lead to new approaches for tissue regeneration.

## 2 Results

The assembly of the FaSt‐Light apparatus is illustrated in Figure 1. The projection system in the apparatus comprises of a light engine (i.e., laser modules), a DMD, and a set of telescopic lenses before and after the DMD. The light engine for the three laser modules (continuous wave emission at 405, 450, and 520 nm) is connected to a single output fiber, which allows homogenization of the laser light beam at each wavelength and a consistent projection into the first telescopic lens set. The first telescopic lens expands the light to be able to cover the entire mirror array in the DMD. The image is then shaped using the DMD and then travels through the second set of telescopic lenses with an iris in‐between the lens to remove the auxiliary images arising from the DMD. The projected image is then directed through the image guide fiber bundle, coupled to the projection setup, into the photoresin.

For the photocrosslinking schemes used in this work, we used 5% w/v gelatin methacryloyl (GelMA) in phosphate buffered saline (PBS) as the base material, for it is one of the most widely used photoresin in light‐based biofabrication.^[^ ^39^, ^40^, ^41^ ^]^ We further used three different photoinitiation mechanisms which have absorption maxima near each wavelength. For photocrosslinking at 405 nm, we used the Norrish Type‐I photoinitiator LAP, which undergoes a homolytic cleavage upon light absorption, producing free radicals which lead to covalent bonding of the methacrylate groups in the resin.^[^ ^42^ ^]^ For 450 nm, we used a redox‐based Norrish Type‐II photoinitiation system containing ruthenium(II) complex (Ru) and sodium persulfate (SPS).^[^ ^42^ ^]^ Here upon photoabsorption, the excited ruthenium(II) complex (Ru(bpy)₃^2^⁺) transfers an electron to sodium persulfate (SPS), generating sulfate radicals and initiating the photocrosslinking process. Notably, this photoinitiation system can result in covalent binding of the methacrylate groups, and the native tyrosine groups in the gelatin.^[^ ^43^ ^]^ Finally, for crosslinking at 520 nm, we used a Norrish Type‐II initiation system consisting of Eosin Y (EY), Triethanolamine (TEOA), and N‐vinylpyrrolidone (NVP).^[^ ^42^ ^]^ In this system, photoabsorption at 520 nm leads to excitation of EY, followed by electron abstraction from TEOA to create amine radicals which initiate the crosslinking process. Here, NVP acts as a chain transfer agent and a reactive diluent, accelerating the crosslinking process and enhancing the overall polymerization efficiency.

**Figure 2:**
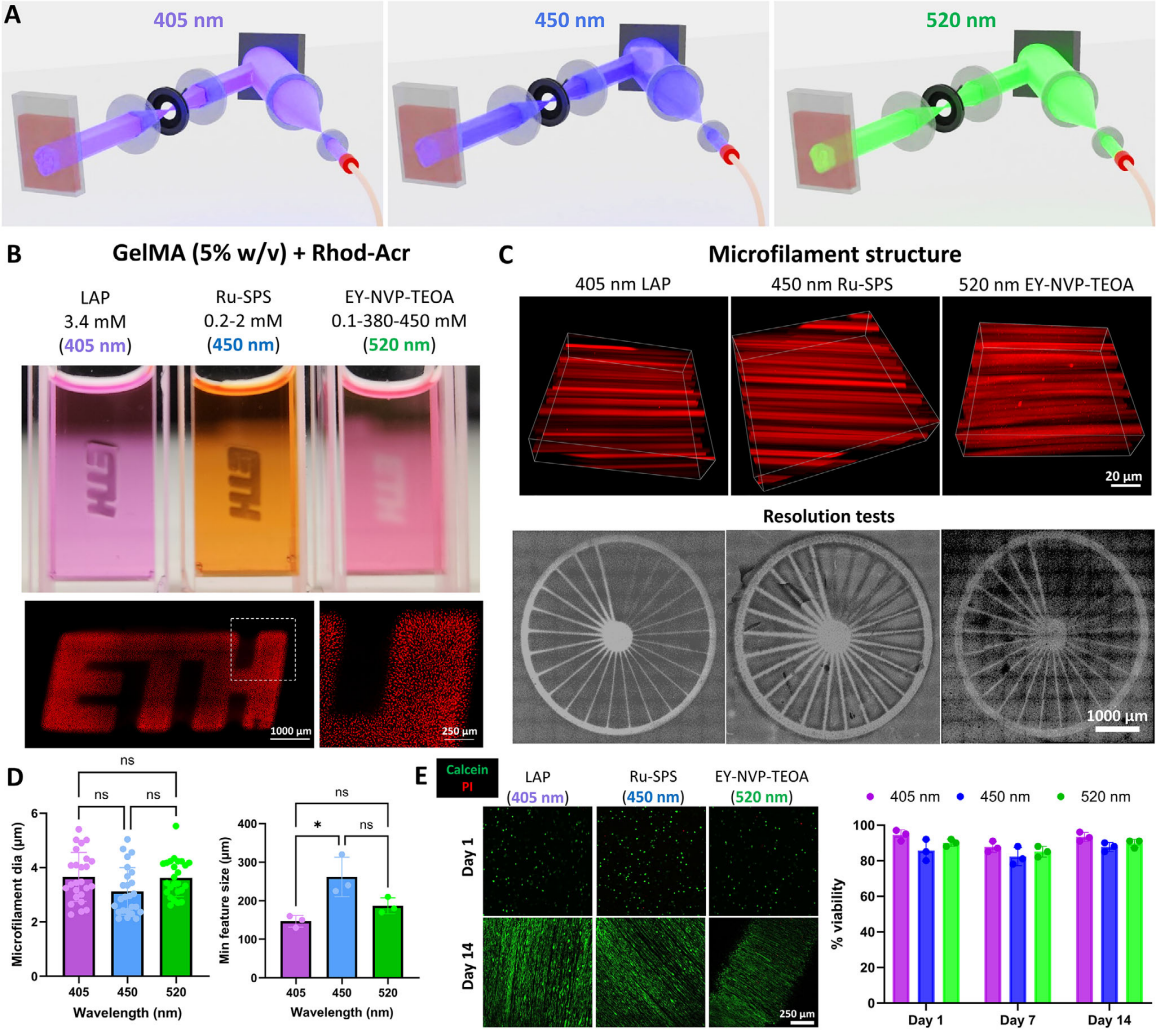
Validation of printing through the multi‐wavelength structured light projection system. A) Illustration of laser activation to derive multiple wavelength projections (405, 450, 520 nm) into a photoresin cuvette. B) Crosslinked GelMA under various photoinitiation systems which have absorption maxima in the different wavelengths. The ETH logo demonstrates microfilaments generated due to optical modulation instability arising from the interaction of the light with the resin. C) Micrographs of the filament structures within the crosslinked constructs and the printed constructs for resolution tests. D) Results of microfilament diameter within the crosslinked constructs and minimum achievable feature size. *p < 0.05. E) Representative images of the viability of C2C12 cells (live cells are stained green with calcein AM and dead cells are stained red through propidium iodide; selected images are shown for days 1 and 14 within the crosslinked constructs) and analysis of viability for each photoinitiation system.

We first conducted tests on the projection system alone (illustrated in **Figure** **2**A) without the image guide fiber bundle, to determine the light doses for crosslinking of GelMA at each wavelength. The resulting prints of ETH logo using the projection system at each wavelength (optimal light doses shown in Figure S1, Supporting Information) and the concentration of the photoinitiators used therein are shown in Figure 2B. The resulting constructs made at each wavelength featured microfilaments generated by optical modulation instability due to the optical autocatalysis (i.e., self‐focusing) of the speckled laser light beam within the photoresin.^[^ ^17^, ^22^ ^]^ The stitched micrograph of the ETH logo (Figure 2B) shows the sectional view of the microfilaments, while a 3D micrograph at each wavelength is shown in Figure 2C. While the photoinitiation mechanisms and the light doses for polymerization (Figure S1, Supporting Information) were different for each wavelength, we did not observe differences in the microfilament diameter (typically 2–6 µm, Figure 2D), which can be attributed to the use of a single fiber optic cable connected to the output of the light engine. We also projected spoke wheel patterns (Figure 2C) within cuvettes to determine the minimum attainable feature size when crosslinked at each wavelength. Here, while the photoresins containing LAP (i.e., 405 nm) and EY‐NVP‐TEOA (i.e., 520 nm) demonstrated similar resolution (150–200 µm), the resins containing Ru‐SPS (i.e., 450 nm) showed larger minimum feature sizes (200–310 µm). This reduced resolution in the Ru‐SPS crosslinked GelMA could be attributable to the strong oxidation potential of the photoinitiation system which, in addition to the methacrylate crosslinking, can lead to the formation dityrosine bonds in the gelatin backbone,^[^ ^43^, ^44^ ^]^ thereby inducing nonspecific crosslinking in the matrix and affecting the resolution. While the above three photoinitiation strategies have been shown to be cytocompatible,^[^ ^22^, ^42^ ^]^ we conducted additional viability tests with our system. For these experiments, we encapsulated myoblasts (C2C12; 10^6^ cells mL^−1^) in separate formulations of GelMA with the three different photoinitiation systems and biofabricated cylindrical constructs by projecting circular images (ɸ = 750 µm) into 2 mm path length cuvettes at each wavelength. The viability of the cells at days 1, 7, and 14 was >80% in each condition (Figure 2E), thereby demonstrating the cytocompatibility of the light doses and the photoinitiation system used in the projection apparatus.

**Figure 3:**
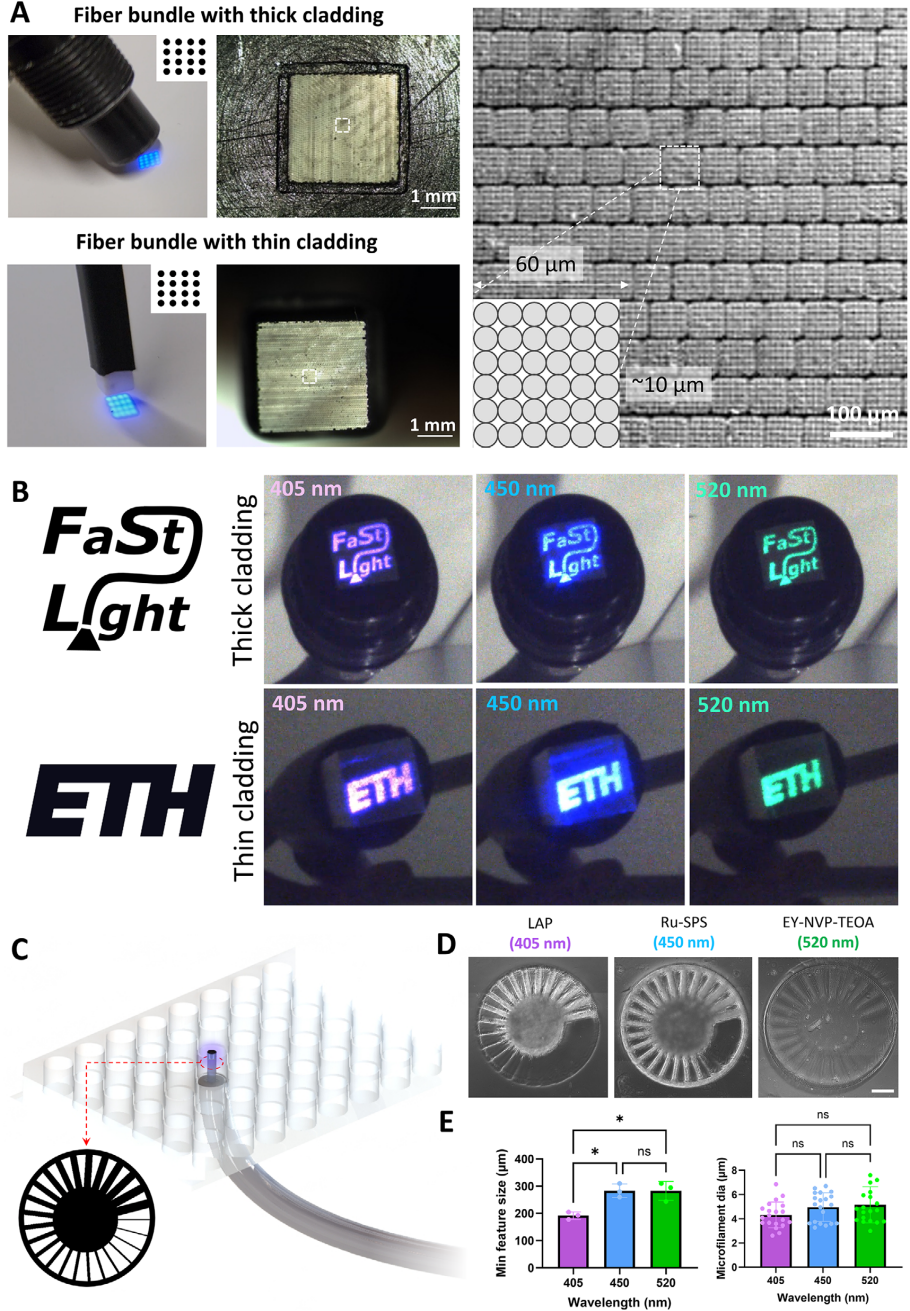
Different types of image guide fiber bundles in the FaSt‐Light apparatus. A) Fiber bundles with thick and thin cladding. Inset images (dotted rectangles) show the corresponding building blocks of the fiber arrays. B) Different images (FaSt‐Light and ETH logos) projected at different wavelengths. C) Fiber bundles being used for bottom‐up projection of spoke wheel image (Φ = 3 mm) into the resin contained within 48‐well plates. D) Crosslinked spoke wheel constructs made at each wavelengths (scale bar: 500 µm). E) Minimum attainable feature size (i.e., spoke width) and microfilament diameters at each wavelength. *p < 0.05.

For subsequent studies, we used the FaSt‐Light apparatus, in which the image guide fiber bundle was coupled to the multi‐wavelength projection setup. **Figure** **3**A shows two different fiber bundles—one with thick cladding and one without—each featuring a 3 × 3 mm^2^ projection cross‐section. Here, a thick cladding can offer enhanced protection and handleability to the fiber (e.g., resin crosslinking onto open tissue sites), while a thin cladding offers smaller form factor (e.g., suitability for endoscopy applications). Each of these fiber bundles feature stacked blocks of fiber arrays, where each block is a 6 × 6 grid of individual fibers. Here the arrangement of the blocks at the input of the fiber bundle matches that at the output, which is necessary for conveyance of the image features through the fiber bundle. Notably, the diameter of individual fibers (ɸ = 10 µm) in the fiber bundle is larger than pitch of the micromirror array (5.5 µm) in our projection setup, which limits the pixel size of the image projected through the fiber bundle to 10 µm. Figure 3B shows images (FaSt‐Light and ETH logo) at each wavelength being projected using the two different fiber bundles (also see Videos S1 and S2, Supporting Information, of the image projection at each wavelength from the two fiber bundles).

To fabricate the constructs for resolution measurements, we filled GelMA resin (100 µL per well; corresponding to ≈1.34 mm height) in 48‐well plate and projected spoke wheel images (Φ = 3 mm) from fiber bundle positioned underneath the well plates (illustrated in Figure 3C). Of note, light intensity attenuates as it travels through the fiber bundle. For instance, the light intensity at the output of the projection apparatus (i.e., the input of the fiber bundle) at 405, 450, and 520 nm was 31.1, 34.5, and 40 mW cm^−2^, respectively. While the power at the output of the fiber bundle (3 × 3 mm^2^ section and 75 cm long; with thick cladding) for 405, 450, and 520 nm was 14.1, 16.7, and 22.5 mW cm^−2^, respectively. Accordingly, we increased the exposure duration of the different GelMA formulations at each wavelength to achieve the same light dose (in mJ cm^−2^; Figure S1, Supporting Information) for material crosslinking. The spoke wheel prints at each wavelength within the 48 well plates are shown in Figure 3D (actual setup is shown in Figure S2 (Supporting Information); also see Video S3 (Supporting Information) for the spoke wheel projections at each wavelength from the fiber bundle), and the corresponding analysis of the minimum attainable feature size and the microfilament diameters are shown in Figure 3E. While the microfilament diameters in the three different photoresin formulations crosslinked by the fiber bundle (images of microfilaments are shown in Figure S3, Supporting Information) were not different, the minimum attainable feature size for the resins containing LAP (i.e., 405 nm) was the lowest (i.e., highest resolution), while the resins containing Ru‐SPS (i.e., 450 nm) and the EY‐NVP‐TEOA groups demonstrated the higher feature sizes (i.e., lower resolution). The reduced resolution in resins containing Ru‐SPS can be attributable to the increased tyrosine crosslinking (similar to Figure 2D), the reduced resolution in resins containing EY‐NVP‐TEOA can be attributed to the prolonged exposure times (71 s) required by the resin formulations. In contrast, the resins containing LAP or Ru‐SPS only needed 26 or 10 s for the fiber‐assisted crosslinking of the resin, respectively. A prolonged exposure can also lead to more nonspecific crosslinking due to the diffusion of free radicals within the photoresin, thereby affecting the resolution. Despite the prolonged exposure needed for the formulations containing EY‐NVP‐TEOA, it had the lowest elastic modulus (≈0.9 kPa; see Figure S4, Supporting Information), whereas the moduli for formulations containing Ru‐SPS and LAP were comparable (≈2 kPa). For all subsequent tests, we only used the resin formulations containing LAP as they resulted in the highest resolution amongst the three formulations (Figure 3E). Also, due to the low modulus, the EY‐NVP‐TEOA (i.e., 520 nm) formulation had demonstrated high shrinkage (> 60% reduction in cross‐section area) in the constructs during the viability tests (Figure 2E), which made these formulations less suitable for later cell infiltration and tissue maturation studies. Furthermore, despite the faster print times enabled by the resin formulations containing Ru‐SPS, the formulations containing LAP are simpler (i.e., a single photoinitiator component) and have lower attenuation coefficient,^[^ ^2^, ^22^ ^]^ resulting in greater penetration of the light into the photoresin. To characterize the depth of crosslinking, we only compared the resin formulations containing LAP or Ru‐SPS (Figure S5, Supporting Information), as these formulations resulted in similar elastic moduli. In these tests, we projected custom images using the image guide fibers into 10 mm path length cuvettes. Here, while photocrosslinking was achieved up to 9 mm depth in the formulations containing LAP, those containing Ru‐SPS only demonstrated photocrosslinking only up to 2.5 mm in depth (Figure S5, Supporting Information). In these tests, the optical self‐focusing effect, where the light is preferentially guided into the crosslinking polymer due to changes in refractive index,^[^ ^17^, ^22^ ^]^ contributed to a reduced scattering of light and maintenance of image features through the crosslinked constructs.

In addition to different types of cladding, the FaSt‐Light apparatus can also deploy different sizes of fiber bundles to accommodate the projection of larger images into the photoresins. For instance, we compared two different sizes of fiber bundles—3 × 3 and 4 × 4 mm^2^—coupled to the projection apparatus (Figure S6A, Supporting Information). While the overall sizes of the bundles were different, the constitutive blocks of fiber arrays in the two bundles were the same (representative image in Figure S6A, Supporting Information), but the number of such blocks is larger in the larger fiber bundle. To compare the effects of the different sizes of fiber bundles, we projected the same spoke wheel images (ɸ = 3 mm) using the two fibers within 48‐well plates as previously shown in Figure 3C. As discussed previously, only photoresin containing LAP was used in these experiments. The representative print and the microfilament distribution within the crosslinked photoresin using the two different fiber bundles are shown in Figure S6B (Supporting Information). The corresponding analysis of the print resolution and the microfilament distribution (Figure S6C, Supporting Information) revealed no difference between the two fiber sizes. This is expected, since the constitutive blocks of the fiber arrays within the two bundles are of the same size. Therefore, the pixel size of the projected images (same as the size of individual fibers; ɸ = 10 µm) are the same through the two fibers.

**Figure 4:**
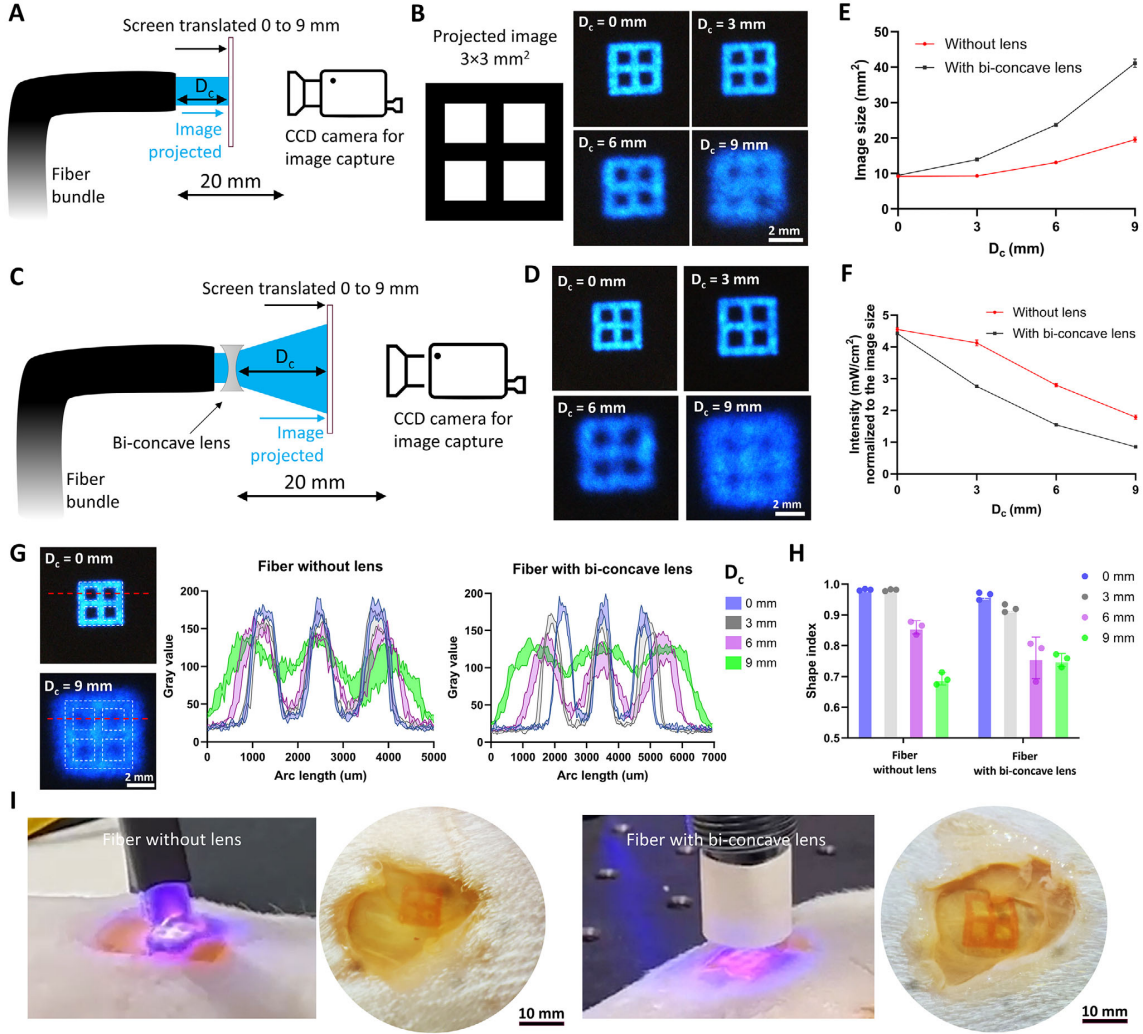
Characterizing the loss in resolution in the images projected from the fibers with or without bi‐concave lens. A) Setup used to capture the images projected from the fiber bundle onto a screen at different image capturing distances (*D*_c_ = 0–9 mm). The distance between the output of the fiber bundle and the camera is fixed to 20 mm. B) The projected images of a 3 × 3 mm^2^ window pattern through the fiber. C) Setup to capture images projected at different distances from a fiber coupled with a bi‐concave lens. D) Projected images from the lens‐coupled fiber. E) Size of the image projected from the fiber. F) Intensity normalized to the size of the projected image from the fiber. G) Estimation of the gray value along the red dotted line in the projected image. White dotted line represents the outline of the ideal projection image. H) Shape index of the projected image when compared to the intended image (after accounting for the expansion from the lens), at different distances from the fiber without or with the bi‐concave lens. I) Demonstration of the use of fibers for projecting images onto resin filled in a skin defect on rat cadavers. Here, the fiber without or with bi‐concave lens was held at a distance of 3 mm from the resin‐filled defect and the window‐shaped pattern was projected onto the resin. The camera images show the crosslinked resin after washing.

In applications involving imaging in hard‐to‐reach regions (e.g., endoscopy), the fiber bundles are typically located a few mm or cm away from the target object to allow effective imaging and avoid contact with the object.^[^ ^35^, ^36^ ^]^ In these applications, custom lens arrangements are often coupled to the fiber bundles for capturing a wider area.^[^ ^35^, ^36^ ^]^ Accordingly, for in situ printing applications, we posit that the fibers will be located a few mm to cm away from the photoresins, and custom lenses could be used for projecting wider images onto the resins. Therefore, we observed the features of the projected images at different distances from the fiber bundles to characterize any loss in resolution. We also compared a bi‐concave lens (diopter = −111.1 m^−1^; half‐angle divergence = 16.7°) coupled at the output of the fiber bundle to be able to widen the projected image. For these experiments, a white screen (0.1 mm thick) was moved away from the fiber bundle and a charge coupled device (CCD) camera was positioned at the opposite end to capture the images being projected into the screen (illustrated in **Figure** **4**A). The images captured at different distances from the fiber without the bi‐concave lens have been shown in Figure 4B, and those with the coupled bi‐concave lens (setup illustrated in Figure 4C) have been shown in Figure 4D. While the projection at the output of the fiber remains collimated up to 3 mm away from the fiber (i.e., the image size and features remain the same), the image gradually expands and loses its features as the distance increases (Figure 4E). In contrast, the bi‐concave lens allows expansion of the projected image, but the image features are only maintained up to 3 mm away from the fiber (Figure 4E). In both conditions (i.e., with or without bi‐concave lens), the intensity of the image normalized to the surface area also gradually decreases (Figure 4F). We further plotted the variations in the gray value of the intensities across a section of the image at different distances (Figure 4G). Here, for both the fiber bundle without or with bi‐concave lens, the distance between the consecutive maxima (and minima) of the gray values increases after 3 mm, showing the increased distribution of light intensities as the distance increases more than 3 mm. Plotting the gray value distribution allowed us to quantitatively assess the shape index of the images, where the distance between the maxima was divided with the feature size of the image had it been a lossless projection (additional details in methods). The shape index results (Figure 4H) also support the qualitative assessments, where the index is above 0.9 (i.e., a high fidelity projection) for up to 3 mm distance from the fiber, but decreases as the distance progresses. As a potential application, we show that the images can be projected from a distance of 3 mm onto resin (GelMA with LAP) filled into skin defects (≈1 mm deep) in a rat cadaver. The FaSt‐Light projection and the corresponding high‐fidelity prints of the window pattern onto rat skin defects are shown in Figure 4I (also see Videos S4 and S5, Supporting Information). Here, when fiber with or without lens was used, the duration of the projection was fine‐tuned to be able to achieve a cumulative light dose of 370 mJ cm^−2^ (optimal for LAP; Figure S1, Supporting Information).

**Figure 5:**
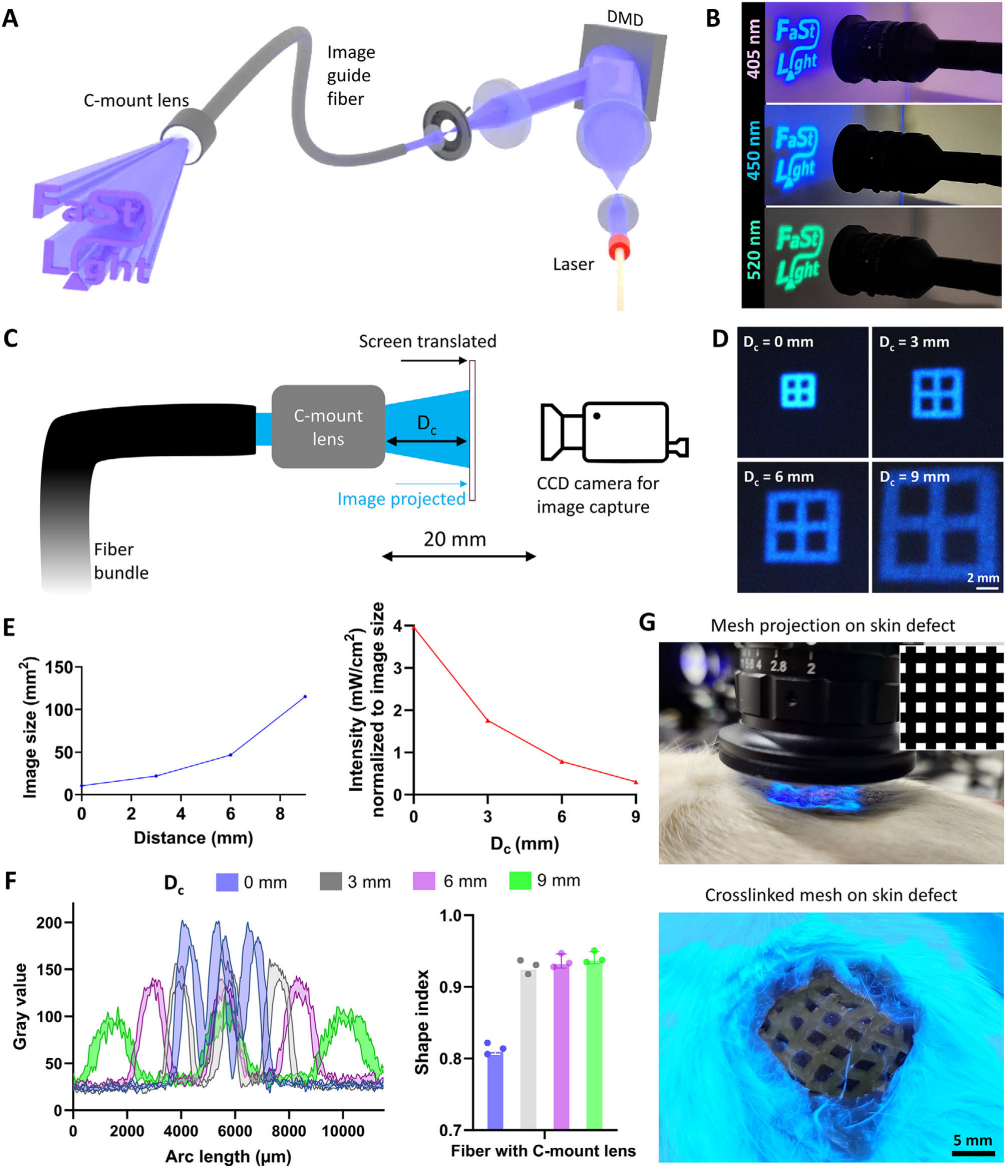
Demonstration of high fidelity image projection using fiber bundles. A) Concept image of coupling of C‐mount lenses onto fiber bundles to preserve image fidelity. B) Projected FaSt‐Light logo at each wavelength at a distance of 20 mm away from a white screen. C) Setup for capturing the images projected onto a screen from a fiber coupled with C‐mount lens. D) Images projected onto a screen at varying distances (0–9 mm) from the lens. E) Variation of the image size and the light intensity normalized to the image size at varying distances from the lens. F) Variation of the gray values at increasing distances from the C‐mount lens and the corresponding shape index when comparing the projected images to the intended image after accounting for the expansion from the lens. G) Crosslinking of resin (inset in the top panel shows the 5 × 5 mm^2^ mesh image which was used) over a rat skin defect. The bottom panel shows the crosslinked mesh (GelMA with LAP was crosslinked at 405 nm and later washed using PBS) over the skin defect.

While the above tests demonstrated that the fiber bundles could be used either standalone or coupled with a bi‐concave lens to project images onto the resin, the distance of the fiber from the resin was limited to a maximum of 3 mm to achieve a high‐fidelity projection. However, for applications where larger area needs to be covered (e.g., tissue defects spanning several cm), a small distance limits the area of the resin which can be effectively crosslinked using a single projection. Here, we demonstrate that a larger projection and resin crosslinking could indeed be achieved by coupling with fiber bundle with a C‐mount lens (**Figure** **5**A), which allows one to preserve the fidelity of the projected image. C‐mount lenses consist of a custom assembly of optical components which enable high‐resolution imaging in scientific and industrial applications.^[^ ^45^, ^46^ ^]^ Figure 5B shows high fidelity image projection, from the C‐mount lens coupled fiber bundle, for all three wavelengths at a distance of 20 mm from the screen. To compare the projection image size and fidelity with the previous results in Figure 4B, we projected the window‐shaped image (3 × 3 mm^2^) and translated the screen from 0 to 9 mm (Figure 5C). The corresponding projection images at the different distances from the C‐mount lens are shown in Figure 5D. Similar to the bi‐concave lens, the C‐mount lens allows expansion of the images, and hence a reduction in the normalized light intensity (Figure 5E). However, unlike the bi‐concave lens, the shape fidelity of the projection image is maintained, which is evident from the results of the gray value profiles and corresponding analysis of the shape index when comparing the actual image to the intended image (Figure 5F, details in methods). Here, the shape index is >0.9 for distances greater than 0 mm, which is highly desirable as most applications will require the fiber bundles to be located away from the target resin. To demonstrate the use of C‐mount coupled fiber bundle, we created a 15 mm wide skin defect (≈1 mm depth) in a rat cadaver and positioned the C‐mount lens 9 mm above the skin defect. Referencing the image expansion from Figure 5D, a 3 × 3 mm^2^ projected image expanded to ≈9 × 9 mm^2^ (i.e., a 3× magnification) at a projection image at a distance of 9 mm. Accordingly, we projected a 5 × 5 mm^2^ meshed image from the fiber bundle to be able to cover the entire skin defect (Figure 5G). After filling the defect with the photoresin (GelMA with LAP), we projected the meshed image pattern from the fiber bundle. Here, as the intensity lowered due to the expansion of the projected image, the exposure duration was adjusted to achieve the same light dose required from crosslinking (similar to the experiments shown previously in Figure 4I). The corresponding crosslinked mesh after washing the defect region with PBS is shown in Figure 5G (lower panel). In the discussion section, we have highlighted the potential regenerative applications of the current state of the C‐mount‐coupled FaSt‐Light apparatus, and further discussed potential approaches to reduce the form factor of the lens‐coupled fibers, to facilitate minimally invasive image delivery for crosslinking resins over hard‐to‐reach tissues, in an in vivo application.

**Figure 6:**
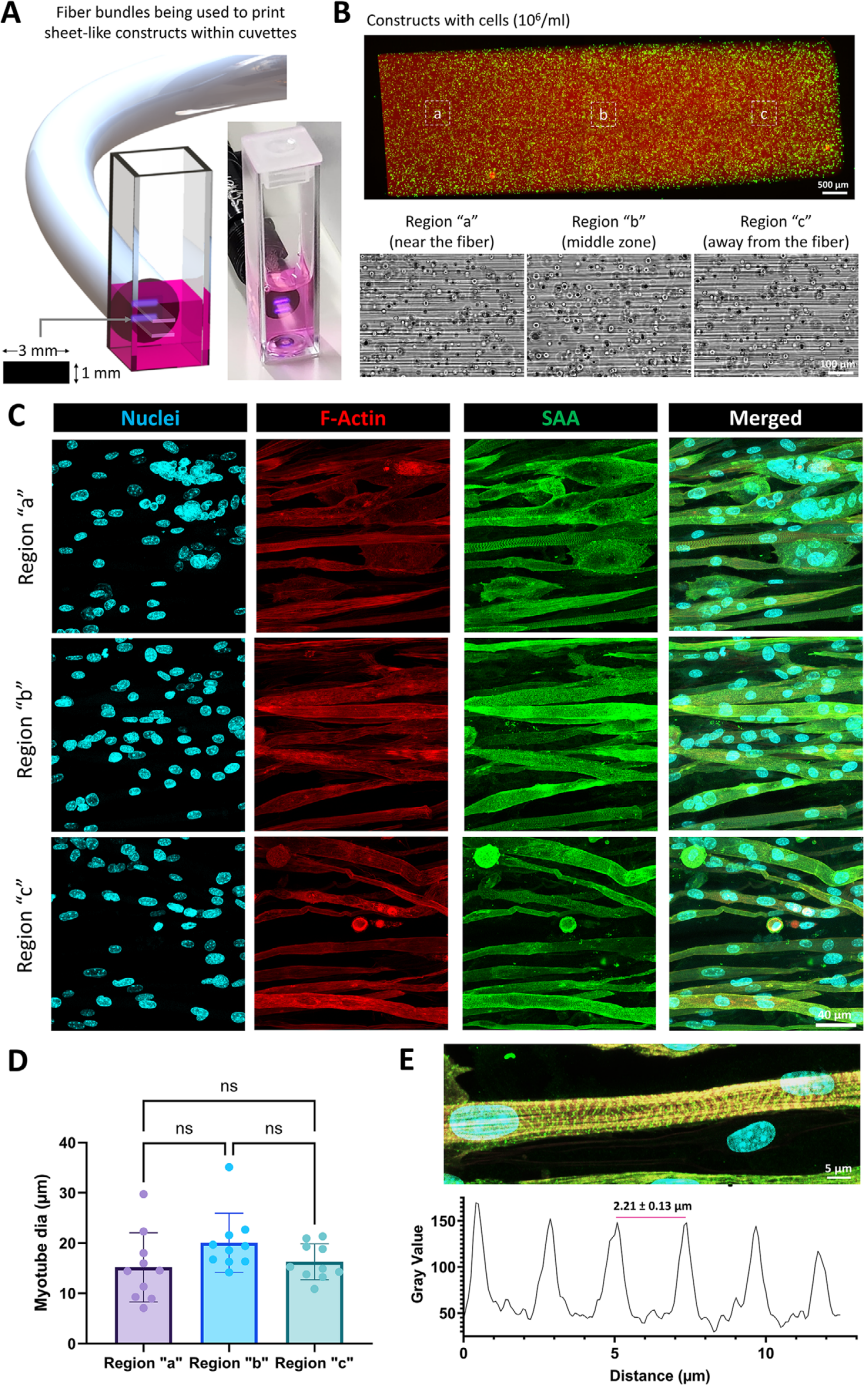
Use of Fast‐Light projection for fabrication of muscle constructs. A) The muscle constructs were fabricated by projecting rectangular images (1 × 3 mm^2^ at 405 nm) from image guide fibers in contact with 10 mm path length cuvettes containing the photoresin (GelMA + LAP). The photograph used rhodamine‐labeled GelMA to allow better visualization. B) 10 mm long sheet constructs with encapsulated C2C12 cells (labeled green using Calcein AM dye; red portion is the rhodamine‐labeled GelMA). The images for the insets (“a”, “b”, and “c” regions) are shown in the bottom, which demonstrate the presence of microfilaments around the cells in each region. C) Matured constructs (1 week of growth + 1 week of differentiation) exhibit key muscle sarcomeric alpha actin staining. Scale bar is 40 µm. D) The myotube diameter is not different in the three regions of the constructs. E) Regularly interspersed sarcomere structures are observed in the constructs with an average sarcomere spacing of 2.21 µm.

Given that the fiber bundles allow maintenance of the light structure (macroscopic image and microscopic speckles), the FaSt‐Light apparatus can be an effective tool for the in situ biofabrication for tissue repair (e.g., defects of muscle, tendon, nerves, etc.). To demonstrate the use case for muscle biofabrication, we encapsulated C2C12 myoblasts at 10^6^ cells mL^−1^ within the GelMA formulations with LAP and transferred the resins within 10 mm path length cuvettes. Of note, in these studies, we had supplemented the resin with iodixanol (a biocompatible refractive index matching agent) to reduce the light scattering effect due to the presence of cells (additional details in methods).^[^ ^12^, ^18^ ^]^ Also, the light dose was increased by 20% to mitigate the effects of light attenuation due to presence of cells. After thermo‐reversible gelation at 4 °C, we used the fiber bundles to project rectangular images (1 × 3 mm^2^) into the cuvettes (**Figure** **6**A). Due to the optical self‐focusing effect, the light projection could crosslink the resin along the entirety of the cuvette, creating 10 mm long rectangular sheets where the microfilaments were present across the entire length (Figure 6B). Since the LAP photoinitiator absorbs light, thereby affecting the light dose along the length of the cuvette, we further characterized the differences in maturation along three regions—region near the fiber bundle during biofabrication, middle zone and away from the fiber bundle. After maturation (1 week of growth + 1 week of differentiation), the muscle constructs (Figure 6C) in each of the three regions exhibited multinucleated myotubes which stained positively for sarcomeric alpha actinin. Notably, we did not observe changes in the three regions with regards to the myotube diameter (Figure 6D) and density (Figure S6, Supporting Information), which is likely due to the short duration of culture to be able to observe region‐specific changes. While the muscle constructs also exhibited sarcomere structures resembling native mouse muscle (≈2.2 µm; Figure 6E),^[^ ^47^ ^]^ the myotube diameters are smaller than those found in native mouse muscle tissues (≈50 µm),^[^ ^48^ ^]^ which is likely due to the short maturation period, low cell densities and absence of other cell types (e.g., fibroblasts, endothelial cells, etc.) supporting muscle maturation.^[^ ^49^, ^50^ ^]^ Longer culture, higher cell densities, and co‐culture systems will be investigated in our future work to achieve biomimetic myotube diameters and other attributes (synchronous contractility, connective tissue deposition, neovascularization, etc.) resembling native muscle tissue.^[^ ^49^, ^50^ ^]^

**Figure 7:**
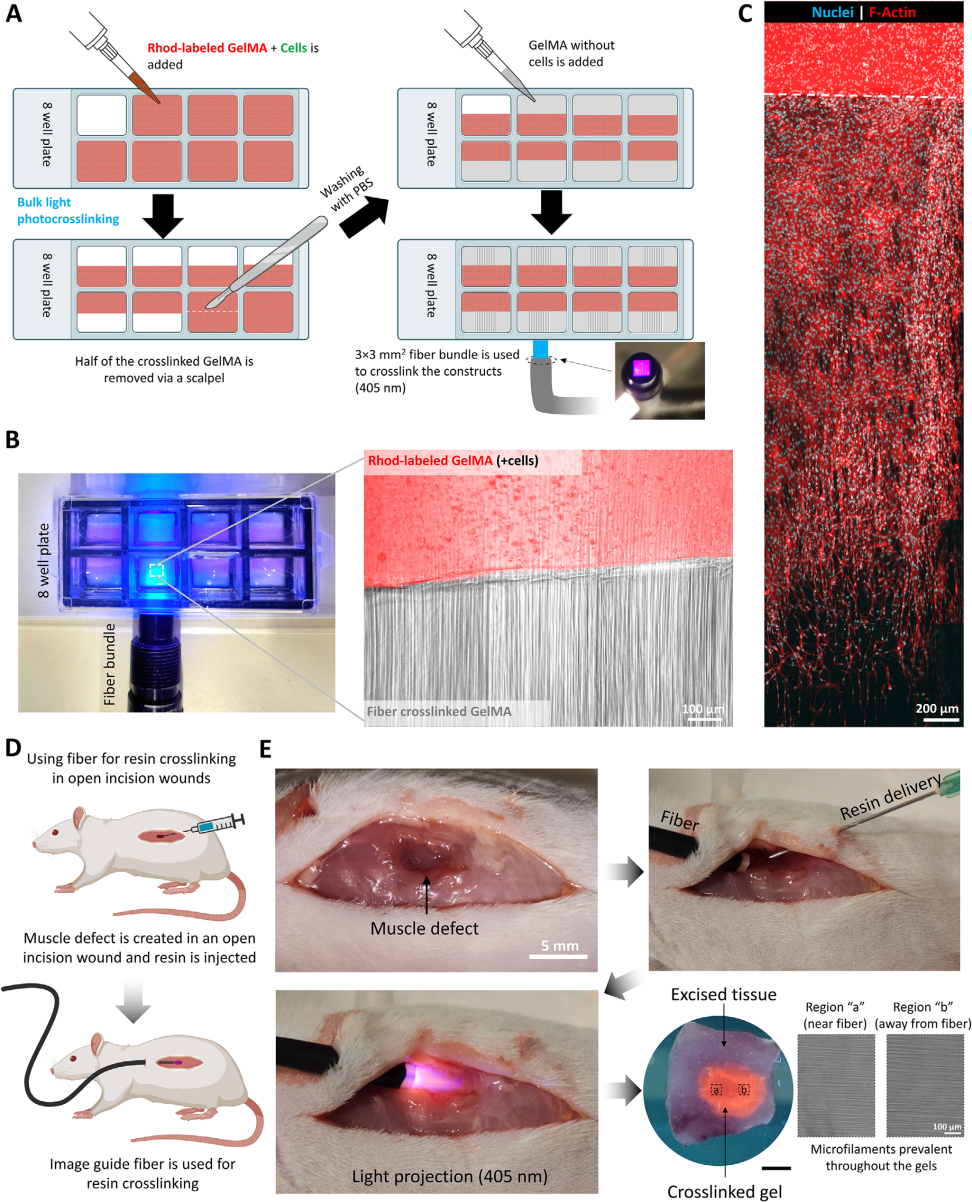
Concept experiments demonstrating cell infiltration into acellular microfilamented matrices, and the use of FaSt‐Light approach for in vivo resin crosslinking. A) Fabrication of the constructs featuring Rhodamine‐labeled GelMA (crosslinked with bulk light exposure to 405 nm in a UV box) containing cells, and acellular GelMA crosslinked with the image guide fiber. B) Camera image (left) of the image guide fiber to crosslink the acellular GelMA to the cellular GelMA and the micrographs showing cellular (rhodamine‐labeled) and acellular (unlabeled) regions of the constructs. C) Stitched micrographs showing cell infiltration into the acellular microfilamented GelMA region 3 d after culture (dotted lines represent the border between the different GelMA regions). D) Concept experiments on the use of FaSt‐Light apparatus to achieve microfilamented resin crosslinking within a muscle defect in rat cadavers. E) Camera images of the muscle defect (incision kept larger than the defect to allow easy visualization), and the insertion of the fiber bundle and syringe needle via separate incisions on either side of the defect. After the light projection, the tissue is excised and imaged. The crosslinked construct is further extracted from the tissue (scale bar is 3 mm) and microstructure (after imaging) shows the prevalence of microfilaments throughout the crosslinked gel.

In addition to in situ biofabrication of cellularized constructs, the fiber bundles can also be used for fabricating acellular biomaterial grafts in situ for new tissue formation and repair. Here, we show that the presence of microfilaments can provide topographic cues for cell guidance and infiltration. As a demonstration, we encapsulated myoblasts (C2C12; 10^6^ cells mL^−1^) in GelMA resins (with LAP; labeled with rhodamine for clear distinction between the layers) and filled the resins within eight‐well plates (**Figure** **7**A) to create a layer height of ≈1 mm (100 µL of resin was added). The resins were then crosslinked using bulk light illumination (405 nm) in a UV box (see methods for details), and a scalpel was used to remove half of the crosslinked cell‐laden hydrogel. The constructs were then thoroughly washed with PBS (to remove any uninitiated LAP), and the excess PBS was removed. In the remaining portion, fresh acellular GelMA was added and crosslinked using the fiber bundle (plain image was projected from the fiber) from the side of the well plate (Figure 7B). The resulting crosslinked constructs (Figure 7B) featured two regions—a rhodamine labeled region with encapsulated cells (bulk light crosslinked), and an acellular region featuring microfilaments (crosslinked using the fiber bundle). Of note, we also observed some microfilaments in the cellular region which was crosslinked with bulk light. This is likely due to diffusion of LAP from the freshly added resin which was then crosslinked with the fiber bundle which introduces the microfilaments. After supplementing media over the crosslinked constructs in the well plates and culturing over 96 h (media changes every 24 h), we observed cell infiltration into the constructs (Figure 7C) up to 3.08 ± 0.41 mm and a predominantly aligned cell morphology throughout the constructs, which demonstrates suitability of the microfilamented constructs in effectively guiding cell infiltration. We further verified whether such an approach could be deployed within an in vivo wound muscle defect in rat cadavers (concept illustrated in Figure 7D). For these experiments, we created an incision in the back of rat cadavers and created a 5 × 3 × 3 mm^3^ defect in the dorsal muscle tissue. Here, a larger incision allowed better visibility of the use of fiber bundle for resin crosslinking. The fiber bundle and a syringe needle were inserted from two separate incisions on either side of the wound site, and the resin dispensed from the syringe followed by blank image projection from the fiber bundle (Figure 7E). Here, the excised tissue allowed a better view of the crosslinked gel in the defect (Figure 7E). When the crosslinked gel was extracted from the tissue site using a spatula and imaged, the microfilaments were prevalent throughout the entire length of the defect. Considering the results from in vitro tests showing cell infiltration, the in vivo proof‐of‐concept experiments demonstrating the fabrication of microfilamented hydrogel scaffolds can open new applications for the regeneration of musculoskeletal defects. In the discussion, we further highlight the future developmental work on the FaSt‐Light approach and the regenerative applications where this approach can be used.

**Figure 8:**
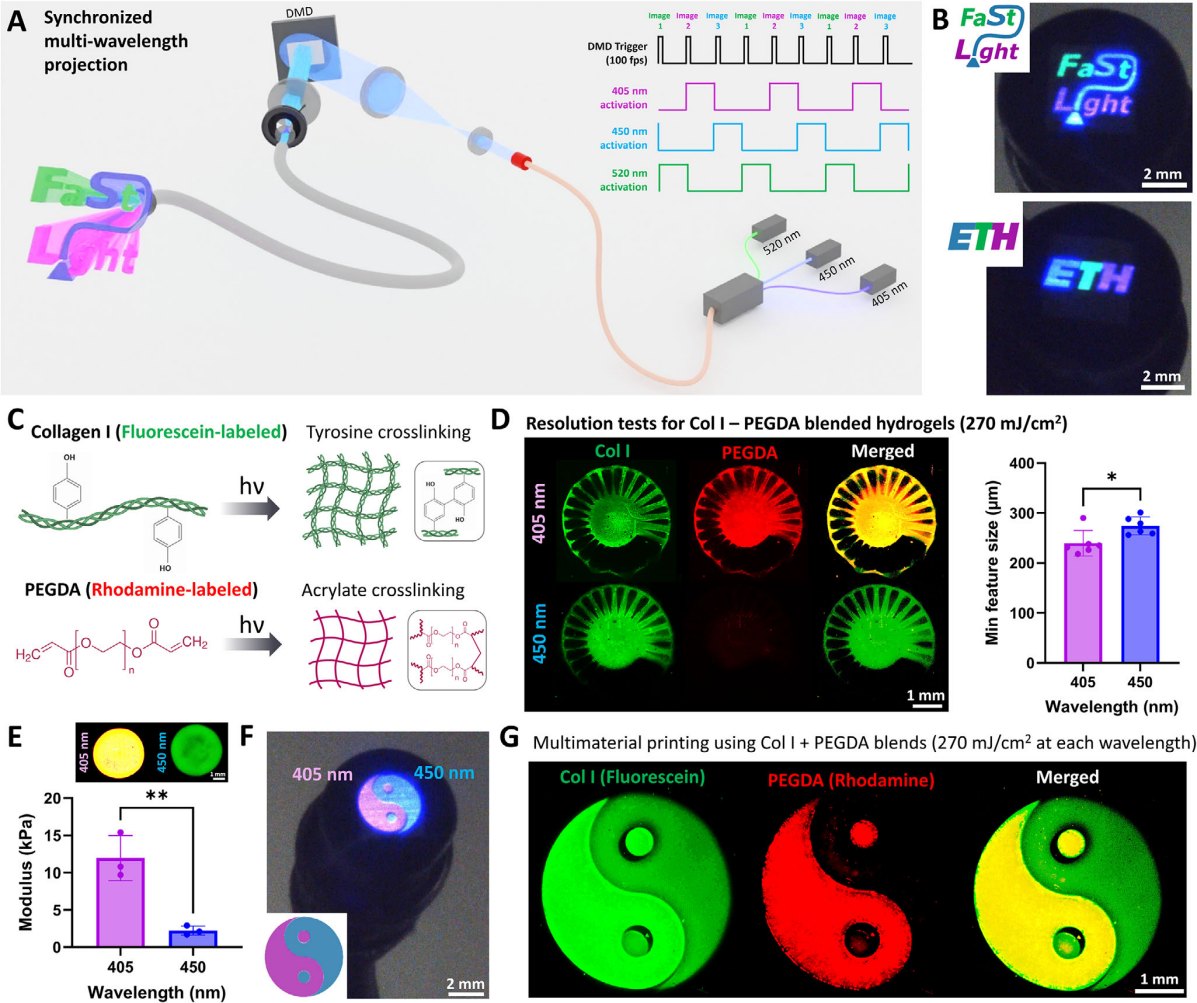
Synergistic multiwavelength FaSt‐Light projection and biofabrication of multimaterial constructs. A) Illustration of laser pulse synchronization with image projections from the DMD to create multiwavelength image projections. B) Projection of FaSt‐Light and ETH logo from the image guide fiber bundles coupled to the multiwavelength projection setup. C) Use of Col I and PEGDA based resin blend which relies on tyrosine and acrylate crosslinking, respectively. D) Resolution tests on the resin blend crosslinked at the optimized light dose (see Figure S8, Supporting Information) which allowed crosslinking of Col I at both wavelengths, and crosslinking of PEGDA only at 405 nm wavelength. The minimum feature size is higher (i.e., lower resolution) in the resin blend crosslinked at 450 nm, which is likely due to the increased swelling of the constructs after washing. *p < 0.05. E) Elastic modulus of constructs (3 mm discs; examples for each wavelength shown on top of each bar) crosslinked at 405 and 450 nm, where the ones crosslinked at 450 nm demonstrated lower modulus due to the presence of only collagen in the crosslinked matrix. Scale bar is 1 mm. **p < 0.01. F) Yin‐Yang pattern projection from the fiber bundles featuring distinct regions with light projection at 405 and 450 nm. G) Crosslinked constructs using the Yin‐Yang pattern, where the regions crosslinked (light dose 270 mJ cm^−2^) at 405 nm featured with Col I and PEGDA crosslinks, while the region crosslinked at 450 nm featured only Col I crosslinking and negligible PEGDA crosslinking.

The aspect of being able to project multiple wavelengths allowed us to demonstrate the use of different photoinitiation systems with the FaSt‐Light apparatus. However, the projections in the studies above were executed one wavelength at a time. Notably, the DMD chip used in the present work has a high frame refresh rate (9523 Hz), which can allow synchronization of the image being projected to the laser wavelength being activated. This approach enables the projection of spatially defined regions with distinct wavelengths within a single projection image. Such a system, when effectively combined with wavelength‐specific photocrosslinking or photodegradation chemistries, can allow in situ photo‐bioprinting or photodegradation of multimaterial constructs.^[^ ^51^, ^52^ ^]^ As a conceptual demonstration of synergistic multi‐wavelength projection, we activated each laser wavelength in pulses synchronized with the image sequence displayed on the DMD (illustrated in **Figure** **8**A). For instance, the images for the FaSt‐Light and ETH logo were divided into three separate images and synchronized with laser activation at 405, 450, or 520 nm, respectively, to create multicolor image projections (Figure 8B). Notably, this strategy leverages control over the DMD image switching rate and its synchronization with laser pulsing to create the perception of a simultaneous multiwavelength projection (when the camera frame rate is lower than the DMD trigger rate; Video S6, Supporting Information) or one‐at‐a‐time flashing projection (when the camera frame is higher than trigger rate; Video S6, Supporting Information).

As a demonstration on the use of the multiwavelength FaSt‐Light projection system to print multimaterial constructs, we created customized resin blends based on pristine collagen I (Col I) and polyethylene glycol diacrylate (PEGDA) containing LAP and Ru‐SPS at a concentration of 3.4 × 10^−3^ and 0.3–0.6 × 10^−3^ m. The formulations were made using off‐the‐shelf components (see the Experimental Section). To allow distinction between the components, the collagen was labeled with fluorescein and the PEGDA labeled with rhodamine (details in the Experimental Section). In this resin formulation, the collagen was susceptible to Ru‐SPS‐initiated crosslinking of the native tyrosine groups,^[^ ^53^ ^]^ while the PEGDA was susceptible to acrylate crosslinking initiated by both LAP and the Ru‐SPS system (Figure 8C).^[^ ^54^ ^]^ Since the Ru‐SPS exhibited a high extinction coefficient at both 405 and 450 nm (peak near 450 nm),^[^ ^55^ ^]^ the collagen in the resin blend could crosslink at both 405 and 450 nm (resin crosslinking response at different light doses is shown in Figure S8A, Supporting Information). Now, to achieve a wavelength‐exclusive crosslinking of PEGDA within the resin blend, we relied on the higher efficiency of the Ru‐SPS‐initiated photocrosslinking in collagen compared to the PEGDA formulations. With low SPS concentration (0.6 × 10^−3^ m) and low light dose (270 mJ cm^−2^), coupled with the negligible extinction coefficient of LAP at 450 nm,^[^ ^54^ ^]^ we were able to achieve constructs with negligible PEGDA crosslinking (i.e., absence of Rhodamine fluorescence) at 450 nm (see Figure S8A, Supporting Information, and the resolution tests in Figure 8D). In contrast, at 405 nm, the constructs exhibited crosslinking of both PEGDA (primarily initiated by LAP) and collagen (initiated by Ru‐SPS). At higher light dose (540 mJ cm^−2^); however, we observed crosslinking of PEGDA within the resin blend at 450 nm (Figure S8A, Supporting Information). Accordingly, we chose 270 mJ cm^−2^ as the optimal light dose at each wavelength to create multimaterial constructs with the resin blend.

The resolution tests using the resin blends at each wavelength and with the optimal light dose are shown in Figure 8D (crosslinking scheme same as in Figure 3C). Here, the minimum feature size was higher (i.e., resolution was lower) for the resin blend crosslinked at 450 nm, which is likely due to the higher swelling observed in the constructs crosslinked at 450 nm compared to those crosslinked at 405 nm (Figure S8C, Supporting Information). This is, in turn, likely due to the reduced number of crosslinks in the constructs crosslinked at 450 nm (featuring predominantly collagen crosslinking and negligible PEGDA crosslinking). The negligible presence of PEGDA in the constructs crosslinked at 450 nm also contributed to the low elastic modulus (≈2.1 kPa) in these constructs (from compression tests performed on discs after swelling; Figure 8E). In contrast, the constructs crosslinked at 405 nm exhibited a substantially higher elastic modulus (≈12 kPa) compared to those crosslinked at 450 nm. The constructs crosslinked at each wavelength also demonstrated presence of microfilaments, which was not significantly different between the wavelengths (Figure S8D, Supporting Information). However, the average microfilament size was higher in the formulations crosslinked at 450 nm, which is also likely due to the increased swelling observed in the formulations (Figure S8D, Supporting Information). Finally, as a demonstration of multi‐material printing, we projected a Yin‐Yang pattern from the fiber bundle (Figure 8F) using the pulsing scheme demonstrated in Figure 8A, and crosslinked the resin blend using the same scheme as Figure 3C. Here, to achieve a cumulative light dose of 270 mJ cm^−2^ the exposure duration was doubled to account for the light pulsation. The crosslinked Yin‐Yang shapes are shown in Figure 8G, where the region exposed to 405 nm exhibited both Col I and PEGDA crosslinking, while the region exposed to 450 nm exhibited predominantly Col I crosslinking and negligible PEGDA crosslinking. In the discussion, we highlight the future scope of this work, and how simultaneous multi‐wavelength projection scheme can be used for in situ biofabrication of complex engineered tissues.

## 3 Discussion

Image guide fiber bundles have traditionally been used for capturing images from hard‐to‐reach regions such as during endoscopy, and to guide the images to camera sensors.^[^ ^56^, ^57^ ^]^ We have demonstrated a reverse approach called FaSt‐Light, where the fiber bundles were used to guide the images generated from a projection system. The approach offers projection of images at multiple wavelengths, allowing the crosslinking of resins along custom shapes, which is not permissible by other approaches which rely on light guide fibers projecting homogeneous light beams.^[^ ^24^, ^25^, ^38^ ^]^ Here, we highlight the current state of development and future scope which could allow the FaSt‐Light approach to develop into a highly scalable and flexible apparatus for resin crosslinking, potentially transforming the field of minimally‐invasive in situ biofabrication.

For the fiber bundles without or with bi‐concave lenses used in this work, the projected images lost their fidelity for distances greater than 3 mm. Here, the use of C‐mount lenses preserved projected image fidelity for larger distances; however, the associated beam divergence resulted in reduced light intensity which required compensation in terms of prolonged exposure duration to achieve resin crosslinking. Also, the form factor of the C‐mount lenses was large, rendering such a system less suitable for minimally invasive photo‐bioprinting applications. Future applications would benefit from coupling miniaturized C‐mount lenses or development of custom lens apparatus to maintain the image fidelity from the fiber bundles for long‐distance projections.^[^ ^45^, ^46^, ^58^ ^]^ Nevertheless, in its present state, the FaSt‐light setup could be used for resin crosslinking onto open tissue sites such as diabetic chronic wounds,^[^ ^59^, ^60^ ^]^ or over the myocardium (i.e., applications involving direct in situ cardiac patch fabrication) during open heart surgery.^[^ ^61^, ^62^ ^]^ Here, the aspects of microfilaments guiding native cell infiltration, differentiation and new tissue formation would be interesting to study and are a potential future exploration area. In such applications, the resin viscosity will have to be carefully fine‐tuned for ease of application and conformation of the resins to the complex contours and dynamic movements of the underlying tissue (i.e., the resin should not flow away due to the tissue shape or its movements). Since the GelMA resins in the present work undergo thermo‐reversible gelation under colder temperatures (typically <24 °C), the present resin formulations can also be potentially suited to in situ biofabrication in dynamic environments, where one could directly add the resin at a cooler temperature and then quickly photocrosslink it before it liquefies.

Notably, for applications involving side‐ways projection of images into the resins, we observed that the optical self‐focusing effect enables the crosslinking of constructs up to 10 mm in length, which indicates that the fibers as standalone devices could potentially be used for the in situ fabrication of aligned tissue constructs or grafts which can promote cell infiltration. Potential applications include the treatment of volumetric muscle loss or peripheral injury repair, etc.^[^ ^63^, ^64^, ^65^, ^66^ ^]^ However, further investigation is needed as to the extent of the optical self‐focusing effect based on the photoinitiation system. Since LAP has a lower attenuation coefficient compared to other initiation systems such as ruthenium complexes,^[^ ^2^, ^22^ ^]^ it can be better suited for applications requiring larger defects. However, whether LAP‐based resins can allow resin crosslinking for defects spanning several centimeters will be a scope of future investigation. Furthermore, LAP is used primarily with modified resins such as those relying on acrylate, methacrylate, or thiol–ene chemistries.^[^ ^67^, ^68^ ^]^ This, in conjunction with under‐studied immunogenic effects of LAP, affect the translational potential of the resins. In contrast, photoinitiators such as ruthenium complexes (e.g., Ru‐SPS system) or riboflavin phosphate (vitamin B12) can allow crosslinking of natural resins such as collagen or fibrinogen using the native tyrosine or histidine residues.^[^ ^69^, ^70^ ^]^ Such a modification‐free crosslinking approach simplifies the formulations, improves the cost effectiveness and the translational potential of these resins.

In our current work, the prints featured a 2.5D macroarchitecture, i.e., the same cross‐section throughout the constructs. Such a strategy may be effective for in situ fabrication of patches,^[^ ^8^, ^9^ ^]^ or for grafts featuring uniaxial alignment such as nerve conduits,^[^ ^71^ ^]^ but may be limited in matching the macroarchitectures of large‐scale defects. In this case, to compensate for the limited penetration depth of crosslinking and to enable 3D biofabrication (i.e., constructs featuring the changing cross‐section area throughout the constructs), the fiber bundles could be made a part of toolheads which can dispense the photoresins over the tissue site concomitantly with the image projection from the fibers. The fiber can be made to mechanistically traverse (e.g., through a robotically guided toolhead) from one side of the defect to the other for consistent crosslinking throughout the entire defect. Here, photoabsorbers (e.g., tartrazine) would be necessary to prevent over‐crosslinking in the already‐crosslinked regions.^[^ ^7^ ^]^ Alternatively, resin crosslinking in larger defects can also be executed using two fibers from either side of the defect. Here, accounting for the attenuation of light in the resin, the light doses will need to be carefully calibrated to achieve a cumulative light dose at the center of the construct equaling that on the extremities of the constructs, to be able to achieve consistent mechanical properties throughout the crosslinked resin. In fact, a multidirectional projection approach using multiple fiber bundles inserted around the defect can be used to mimic the native multidirectional matrix arrangement (such as in cardiac tissues).^[^ ^16^, ^72^ ^]^ Furthermore, by projecting different images from multiple fibers inserted around the defect, one could achieve a cumulative 3D light dose recapitulating the desired macroarchitecture of the defect site, enabling an approach similar to multi‐direction image projection for volumetric printing,^[^ ^22^, ^73^ ^]^ but performed in situ.

Our work demonstrated that the FaSt‐Light approach can be used for the fabrication of anisotropic muscle tissue constructs or acellular grafts which promote cell infiltration and alignment. We have also demonstrated proof‐of‐concept studies on the in vivo fabrication of patch‐like geometries on wound defects or microfilamented biomaterial grafts within muscle defects in rat cadavers. For the latter study on muscle defects, we created a large incision in the defect site to allow easy visibility of the surgical procedure where the fiber bundle and the resin‐filled syringe were inserted on opposite sides of the defect zone. However, for a future preclinical evaluation in live animal models, such study could be performed minimally‐invasively without the necessity for large incisions. Notably, to investigate printing within deeper areas, e.g., stomach or heart, one would benefit from access to separate endoscopic apparatus and imaging system to view deeper regions of the body.^[^ ^56^, ^74^ ^]^ Such a study would also necessitate an optimization of the resin viscosity and adhesion characteristics to allow temporary adhesion to the contours of the organs,^[^ ^9^, ^75^ ^]^ followed by photocrosslinking using the fiber bundles. Importantly, for these applications, washing off the uncrosslinked photoresin may be crucial to mitigate any unwanted immunogenic effects of the remaining photoinitiators or unreacted monomers.^[^ ^76^, ^77^ ^]^ We aim to investigate these aspects in our future studies by collaborating with veterinary surgeons with established expertise in endoscopy to explore in situ biofabrication within deeper organs.

Analogous to the muscle defect study, the flexibility of the fiber bundles can enable access to anatomically restrained regions of the body, allowing a direct exposure of the resins to the image patterns coming from fiber bundles. This is advantageous over approaches where the resins are delivered deep within tissue (e.g., subcutaneously) and the light is projected from outside the tissue. By utilizing fiber bundles, we could achieve in vivo crosslinking of the resins between the 400 and 520 nm wavelength range (a range where most photoinitiators have high extinction coefficients),^[^ ^2^, ^22^ ^]^ which would otherwise be difficult if we were to use a projection system from outside the tissue. For instance, even with ≈0.64‐mm‐thick skin tissue (derived from nude mice cadavers), the attenuation of the light is >70% at each wavelength (see Figure S9, Supporting Information). Notably, there can be applications where the biomaterial resin may need to be administered inside the tissue (e.g., myocardium),^[^ ^78^, ^79^ ^]^ followed by a spatially controlled polymerization using structured light from the image guide fibers. For such applications, near‐infrared (NIR) wavelengths can minimize light scattering and allow deep penetration within the tissue,^[^ ^80^, ^81^ ^]^ and can be used together with NIR‐sensitive photoinitiators (e.g., upconversion nanoparticles) to crosslink the resins.^[^ ^82^, ^83^ ^]^ As a future concept, we have also demonstrated the use of NIR wavelength projection from the FaSt‐Light apparatus (Figure S10A, Supporting Information). For these studies, the multispectral light engine (i.e., the three‐laser module) was replaced by a NIR laser (780 nm), and custom images were projected from the fiber bundles (Figure S10B,C, Supporting Information). Compared to the wavelengths we have used in this work, the NIR allows substantially higher light transmission through tissues at different thicknesses (Figure S10D,E, Supporting Information), and offers a promising strategy for in situ biofabrication using the fiber bundles. For future studies utilizing NIR sources for printing, the effects of light scattering, when transmitting the images through the tissue, will also need to be investigated and optimized to enable high‐fidelity printing.

As a future concept to be used in multimaterial printing, we also demonstrated a control strategy to allow synergistic multiwavelength projection using the FaSt‐Light apparatus. Notably, different photoinitiation systems such as the ones described in this work have extinction coefficients spanning wide ranges of wavelengths and can be deployed for a wide variety of photocrosslinking mechanisms (e.g., step‐growth or chain‐growth‐based),^[^ ^2^, ^54^, ^55^ ^]^ which complicates the process of achieving wavelength‐selective material crosslinking. In the current work, to allow selective material crosslinking at 450 nm, we leveraged the reaction efficiency of the tyrosine crosslinking of collagen over that of acrylate crosslinking in PEGDA in the presence of low concentration of Ru‐SPS. However, such a multiwavelength printing scheme can be a complex optimization problem for any new resin blend. In particular, the addition of photoabsorbers may be necessary to enhance spatial control over photocrosslinking between regions exposed to different wavelengths.^[^ ^84^ ^]^ Alternately, selective photodegradation can be a robust strategy to achieve multimaterial printing,^[^ ^52^ ^]^ or selective photografting of biomolecules can also be leveraged to spatially alter the biochemical cues available to the cells.^[^ ^19^, ^20^ ^]^ As a facile biofabrication approach featuring single material resins with encapsulated stem cells, wavelength‐selective gene editing using photo‐uncaging of oligonucleotides can also be used to induce spatially‐selective differentiation of cells,^[^ ^85^, ^86^ ^]^ leading to the in situ formation of complex tissues.

In the future, verification and validation of the suitability of FaSt‐Light approach toward applications such as wound healing,^[^ ^87^, ^88^ ^]^ volumetric muscle loss,^[^ ^63^, ^64^ ^]^ peripheral nerve injury repair,^[^ ^65^, ^66^, ^89^ ^]^ etc., would require detailed in vitro characterization studies with multiple cell types and in vivo validation using animal models. For these injury models, careful consideration will be needed in terms of the orientation of microfilaments (i.e., the direction of light projection from the fiber bundles), which will ideally need to match that of the tissue at the distal and proximal ends of defect, to enable effective cell infiltration and matrix deposition which aligns with the native fiber orientation. The stiffness of the in situ photofabricated grafts will also need to be spatially tuned as it can play a crucial role in cell migration, proliferation, inflammatory response, and matrix production.^[^ ^90^, ^91^, ^92^ ^]^ For instance, in tissue models based on tenocytes encapsulated within GelMA matrices,^[^ ^91^ ^]^ we recently demonstrated that the matrix stiffness plays a key role in governing nuclear morphology and mechanical confinement. Here, tenocytes present in stiffer Flight matrix (≈40 kPa) demonstrated more elongated nuclei (higher nuclear aspect ratio) and aligned collagen deposition, while those in lower stiffness (<10 kPa) FLight matrix showed a lower nuclear aspect ratio and misaligned collagen deposition.^[^ ^91^ ^]^ This study has implications for the biofabrication of tissue‐engineered tendon models, offering a platform to better understand tendon physiology and pathology while enabling the evaluation of potential therapeutic strategies.^[^ ^93^, ^94^ ^]^ In another study encapsulating dorsal root ganglions in microfilamented hydrogels made using gelatin‐norbornene (Gel‐NB) and thiol‐functionalized gelatin (Gel‐SH),^[^ ^89^ ^]^ we observed higher neurite outgrowth in soft hydrogels (≈0.6 kPa) compared to stiff gels (≈5 kPa). These findings are relevant for the creation of conduits or grafts for peripheral nerve injury repair, or as tissue models of nerve injury or neuromuscular defects.^[^ ^95^, ^96^ ^]^ In future studies, the multiwavelength projection approach with the resin blends can also enable spatial tuning of the material composition and stiffnesses,^[^ ^97^ ^]^ ultimately leading to the fabrication of complex tissue models or scaffolds to study cell durotaxis and matrix secretion,^[^ ^92^, ^98^ ^]^ which ultimately affects tissue regeneration. Finally, in the future, we envision the integration of the FaSt‐Light system onto mechanized robotic toolheads which can maneuver into hard‐to‐reach regions and precisely position and perform in situ printing of multicellular and multimaterial grafts at the tissue site. Mechanization of the fiber bundle‐assisted printing approach with new advances in deep learning of the images captured during the biofabrication process (i.e., image guided therapy) can further improve the translational potential for an in vivo usage by reducing human errors.^[^ ^57^, ^99^ ^]^

## 4 Conclusion

FaSt‐Light integrates image guide fiber bundles with a multi‐wavelength projection system, offering a new approach for in situ photo‐biofabrication. The images are guided through fiber bundles which come in a variety of shapes and sizes, offering flexibility on the maneuverability, handleability, and the location of fabrication. The use of multi‐wavelength projections allows compatibility with a variety of photoinitiation mechanisms. In addition to a controlled microarchitecture (through controlling the projected image), the resins crosslinked with FaSt‐Light also feature microfilaments (due to optical modulation instability) which allow cell‐guidance and infiltration, opening new applications for in vivo tissue biofabrication or repair. By the coupling of appropriate lenses to the fiber bundles, the image fidelity can be maintained for image projections several centimeters away. The FaSt‐Light approach offers new pathways of research into tools which combine multiple printing modalities, mechanization, and concomitant imaging with printing, potentially ushering in a new era of in situ biofabrication and transitioning away from traditional benchtop devices.

## 5 Experimental Section

### Hardware and Consumables Sourcing

The fiber bundles were procured from Schott AG (Mainz, DE). The lenses and optomechanical components for the FaSt‐Light apparatus, as well as the power meter (S121C; 400–1100 nm measuring range) were procured from Thorlabs GmbH (Bergkirchen, DE). The DMD (evaluation board DLPLCR67EVM) and its controller (DLPLCRC900DEVM) were procured from Texas Instruments. The multi‐wavelength laser light engine was procured from Lasertack GmbH (Fuldabrueck, DE). 10 mm path length cuvettes were procured from Thorlabs GmbH (CV10Q35F). Eight‐well plates for cell infiltration tests were procured from ThermoFisher (Nunc Lab‐Tek II Chamber Slide System; 154534). UV box for fabricating bulk GelMA resins (BSL‐01) was procured from Opsytec Dr. Gröbel GmbH. The digital‐to‐analog converters for trigger input to the DMD were sourced from DFRobot (2‐Channel I2C DAC 0–10 V). The Arduino Uno for laser timing control was purchased from Arduino SA (Chiasso, Switzerland). The NIR laser was a NewFocus 780 (Tunable laser diode TLB 6712, Newport) combined with a tapered amplifier TA‐7613 (Newport).

### Chemical Sourcing

Merck KGaA (Darmstadt, DE) was the source to procure Gelatin (Type B, G6650) and methacrylic anhydride (760‐93‐0), acryloxyethyl thiocarbamoyl rhodamine B (Rhod‐Acr; 908665), Dulbecco's modified eagle medium (DMEM; D6429), Penicillin‐Streptomycin (P/S; P4333), Trypsin‐EDTA solution (T4049), fetal bovine serum (FBS; F1283), horse serum (H0146), propidium iodide (537059), Calcein‐AM (148504‐34‐1), Triton‐X100 (T8787), paraformaldehyde (PFA; 148504‐34‐1), bovine serum albumin (A7030), and phalloidin (P1951). Hoechst (33342) used for nuclear staining was procured from ThermoFisher. The primary antibodies consisted of monoclonal anti‐α‐actinin (Sarcomeric) antibody (SAA) produced in mouse (A7811, Merck). Secondary antibody for SAA was Alexa Flour 488 goat anti‐mouse IgG (A‐11004, Invitrogen). Iodixanol solution (Optiprep) was procured from Stemcell Technologies. Collagen I solution (bovine, 3 mg mL^−1^) was purchased from Advanced BioMatrix (#5005, PureCol). Fluorescein isothiocynate (Pierce FITC Antibody Labeling Kit, 53027) was purchased from ThermoFisher, and PEGDA (Mn 700, 455008) and acrylated rhodamine (Acryloxyethyl thiocarbamoyl rhodamine B; 908665) were purchased from Merck.

### GelMA Synthesis and Constitution

The GelMA was synthesized through a reaction with methacrylic anhydride based on a previously published work.^[^ ^17^ ^]^ The resin was constituted in PBS and the photoinitiator components added (separate formulations for each wavelength) as per the concentrations previously shown in Figure 2. For experiments with cells, iodixanol solution was supplemented instead of PBS to be able to match the refractive index range of cells (1.37–1.375) based on existing studies.^[^ ^12^, ^18^ ^]^ For the tests requiring fluorescence, Rhod‐Acr stock in DMSO (stock concentration at 10 mg mL^−1^) was added at 1 µL mL^−1^ into the resin formulations, similar to a previous work.^[^ ^100^ ^]^

### Muscle Construct Fabrication and Culture

C2C12 murine myoblast line was cultured in growth media consisting of DMEM supplemented with 10% w/v FBS and 1% v/v P/S. The cells were passaged at 80% confluency using Trypsin‐EDTA and counted via an automated cell counter (LUNA‐FX7, Logos Biosystems). The cells were then resuspended in GelMA resins with LAP at 10^6^ cells mL^−1^ (details on optimization of iodixanol concentration are provided in the Experimental Section). The resins were then transferred to 10 mm path length cuvettes and stored at 4 °C for 10 min (to allow thermo‐reversible gelation of the resin) prior to printing. For printing, the fiber bundle was positioned in contact with the cuvette and light projection executed at a light dose increased by 20% to account for light scattering due to cells (optimized from pilot experiments). After printing, the cuvettes were heated at 37 °C and the constructs retrieved, washed with PBS to remove the unreacted resin and transferred to 12‐well culture plates. The constructs were cultured in 2 mL of growth media (media changes every 48 h) up to one week, followed by differentiation in DMEM containing 2% v/v horse serum and 1% v/v P/S.

### Cell Infiltration Tests

C2C12 murine myoblasts were encapsulated in GelMA resins with LAP (without Iodixanol) at 10^6^ cells mL^−1^ within the eight‐well plates (details in the consumables sourcing). 100 µL (≈1 mm construct height) of the resin was added to each well and crosslinked within the UV chamber (details in hardware sourcing) with the same light dose as the fiber‐crosslinked GelMA. Half of the cell‐laden GelMA was then removed using a scalpel and the remaining crosslinked constructs washed with warm PBS (at 37 °C) to remove un‐initiated LAP. Fresh acellular GelMA (at 37 °C) was then added into the empty half of each well plate (i.e., the regions where the cell‐laden constructs were removed from), and the well plates stored at 4 °C for 15 min. The fiber was used to then crosslink the resins (light doses optimized from Figure S1, Supporting Information), and the unreacted resin removed using warm PBS. The constructs were cultured in 150 µL of growth media above the constructs (media changes every 24 h) up to 96 h, followed by fixation (methods above) and staining for nuclei (DAPI) and F‐actin (phalloidin).

### Live/dead Analysis and Immunohistochemistry

The sources of the dyes and antibodies have been listed in the chemicals sourcing section in the methods. Calcein‐AM and propidium iodide were used to stain live and dead cells respectively based on our previous work.^[^ ^17^ ^]^ For immunostaining, the media was removed and the constructs washed in warm PBS, followed by incubation in 4% v/v PFA for 30 min. The PFA was then removed and the constructs thoroughly washed with PBS. The constructs were then incubated in 0.1% v/v Triton‐X100 solution for 30 min at room temperature followed by washing thoroughly with PBS. Blocking was then performed in 5% w/v BSA‐PBS solution for 1 h. Primary antibody was diluted in 1% w/v BSA‐PBS solution as 1:500 and incubation of the constructs with the primary antibody solution was performed in refrigeration conditions (4 °C) for 12 h. The constructs were then thoroughly washed with PBS and then incubated for 2 h in secondary antibody solution consisting of 1:500 dilution of the IgG antibodies and 1:1000 for Hoechst and phalloidin. The samples were then thoroughly washed with PBS and imaged in a Confocal microscope (SP8‐AOBS‐CARS, Leica).

### Shape Index Calculations

The shape index of the images projected onto the screen was calculated by comparing the size of the actual image to the size of the intended image. For this, the distance (arc length) between the first instance of the minimum gray value and the last instance of the minimum gray value along the arc (red line depicted in Figure 5G) was taken as the actual width of the image. The intended image was superimposed onto the actual image after accounting for the divergence of the light from the fiber bundles (with or without the lenses). The shape index (always <1) was the ratio of the width of intended image to the width of the actual image.

### Multiwavelength Projection Setup

The details of the components are provided in the hardware sourcing section in the methods. The image projections were controlled using the software provided by Texas Instruments (DLP LightCrafter DLPC900 GUI). Here, within the “Pattern On‐The‐Fly mode,” the desired image sequence was uploaded, and the exposure duration of each image was adjusted. Wherever needed, the “Repeat” option was activated to allow a continuous loop of the image projection. The controllers for the three lasers in the multispectral light engine were connected to digital‐to‐analog converters for trigger input. The synchronization and precise timing control was realized using an Arduino Uno for signal generation and its I2C protocol for master‐slave configuration connection to the DACs, where the Arduino served as the master and clock reference. The signals passed to the controllers were square waves. Using custom written code, voltage high periods were varied for lasers and DMD to achieve frequencies of up to 100 Hz. Controlling the individual signal amplitudes for the lasers (0.3–5.0 V corresponding to 0% and 100% power, respectively) allowed control of the power output of each laser and normalization of light intensity when required.

### Multimaterial Prints Using Multiwavelength Projection within Resin Blends

The material sources have been provided in the chemicals sourcing section in the methods. The resin blends were prepared by mixing, in DI water, the stock solutions of collagen I (originally 3 mg mL^−1^) and PEGDA 700 (originally 1.12 g mL^−1^) to achieve a final concentration of 2.7 and 51 mg mL^−1^, respectively, as well as the stock solutions of Ru (30 × 10^−3^ m stock), SPS (30 × 10^−3^ m stock), and LAP (2% w/v stock) to achieve a final concentration of 0.3, 0.6, and 3.4 × 10^−3^ m, respectively. For fluorescent labeling, stock solutions of FITC (10 mg mL^−1^ stock in DMSO) and acrylated Rhodamine (10 mg mL^−1^ stock in DMSO) were added at 1 µL mL^−1^ to render fluorescence to the molecules. Here, the FITC conjugated to the primary amines in the collagen matrices, while the acrylated rhodamine conjugated to the PEGDA network during photocrosslinking. For multiwavelength printing, the resin blends were added to well plates and the image projections executed from the fiber bundles positioned underneath the well plate at different light doses. The constructs were washed with milli‐Q water to remove the unreacted resin, and imaging executed via a fluorescent microscope (EVOS M5000).

### Statistical Analysis

For studies with three groups (i.e., when comparing the effects of the three wavelengths or the three regions in muscle models), one‐way Anova with Tukey HSD posthoc tests were used. For studies with two groups (in the multi‐wavelength printing study), unpaired *t*‐tests (two‐tailed) were used for determining the statistical differences. The statical analysis were performed and the graphs were plotted in GraphPad Prism 10.

## Conflict of Interest

The authors declare no conflict of interest.

## Supporting information



Supporting Information

Supplemental Movie 1

Supplemental Movie 2

Supplemental Movie 3

Supplemental Movie 4

Supplemental Movie 5

Supplemental Movie 6

## Data Availability

The data that support the findings of this study are openly available in ETH Research Collection at https://doi.org/10.3929/ethz‐b‐000710181, reference number 710181.
