# Supplementary material for: Structured Light Projection Using Image Guide Fibers for In Situ Photo‐biofabrication
Source: Adv Mater. 2025 Apr 29;37(27):2419350. doi: 10.1002/adma.202419350 (PMC12243700; doi:10.1002/adma.202419350)
Supplement: Supplementary file 1 — Supporting Information [file ADMA-37-2419350-s004.docx]

**Supplemental Information**

**Structured light projection using image guide fibers for in situ photo-biofabrication**

Parth Chansoria^1^, Michael Winkelbauer^1^, Shipin Zhang^1^, Jakub Janiak^1^, Hao Liu^1^, Dimitar Boev^1^, Andrea Morandi^2^, Rachel Grange^2^, Marcy Zenobi-Wong^1^*

^1^ Department of Health Sciences and Technology, Institute for Biomechanics, Tissue Engineering and Biofabrication Group, ETH Zürich, Switzerland

^2^ Department of Physics, Institute for Quantum Electronics, Optical Nanomaterial Group, ETH Zürich, Switzerland

*Correspondence: [marcy.zenobi@hest.ethz.ch](mailto:marcy.zenobi@hest.ethz.ch)

Supplemental information consists of Figures S1 to S10


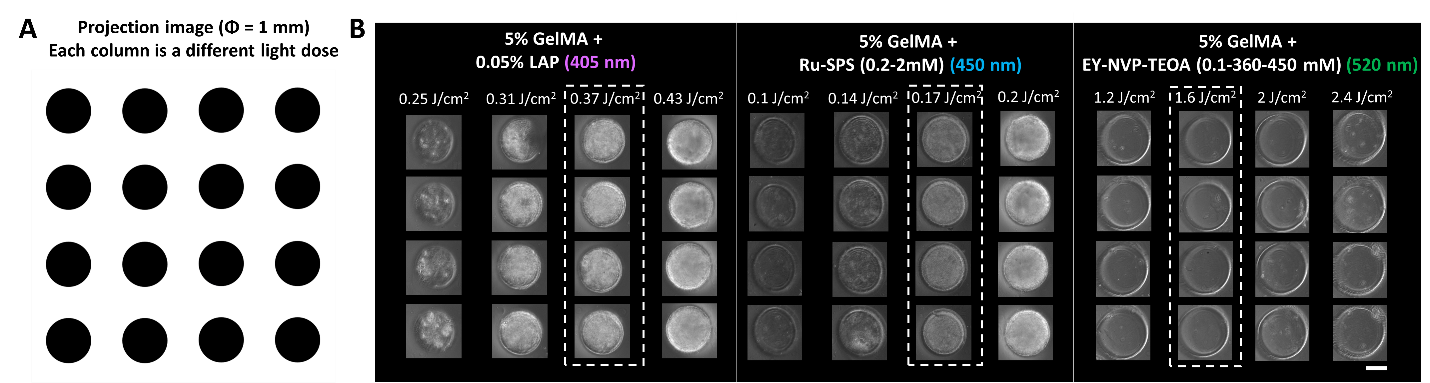


**Figure S1. A.** Projection image for the optimization of the light dose for crosslinking (resins contained within 2 mm path length cuvettes) at each wavelength (photoinitiator components and their components are listed in the images). **B.** Crosslinked images and optimal light doses for each wavelength (marked by dotted rectangles) which result in cylinder diameter closest to the intended design ɸ = 1 mm. Scale bar = 500 µm.


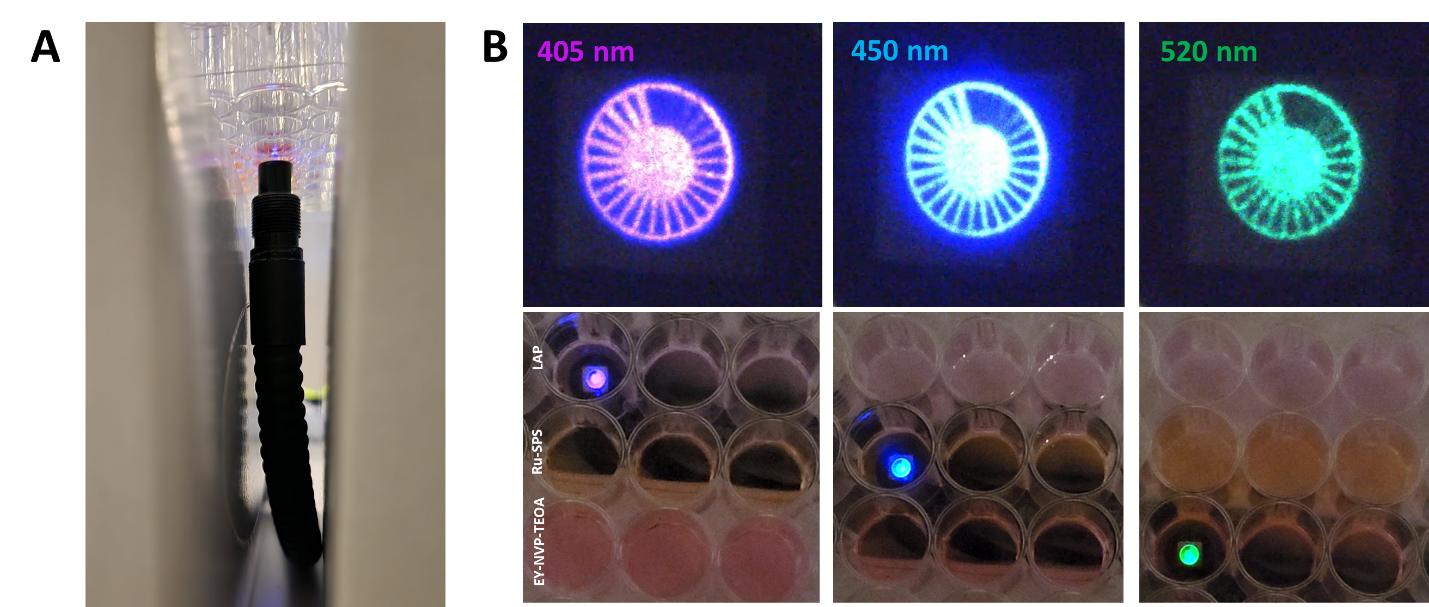


**Figure S2. A.** Setup for the bottom-up projection of images into 48 well plates using the image guide fiber bundles. **B.** Projected spoke wheel patterns at each wavelength within resins (200 µl/well) contained within the well plates.


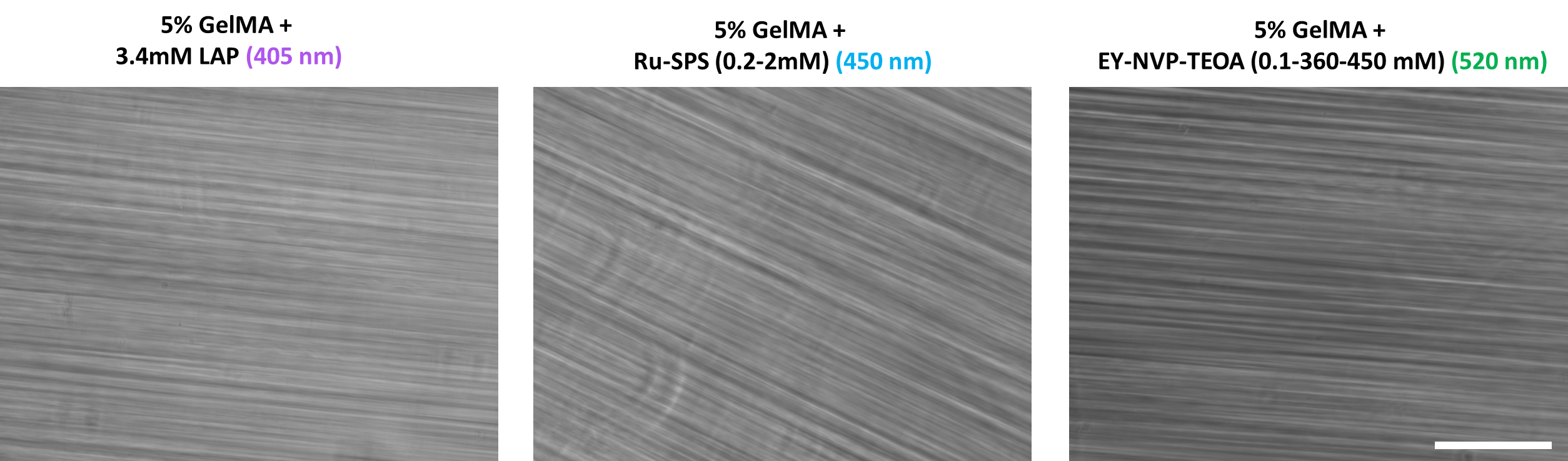


**Figure S3.** Micrographs of microfilament structures within the different photoresin formulations crosslinked using the fiber bundle. Scale bar = 150 µm.


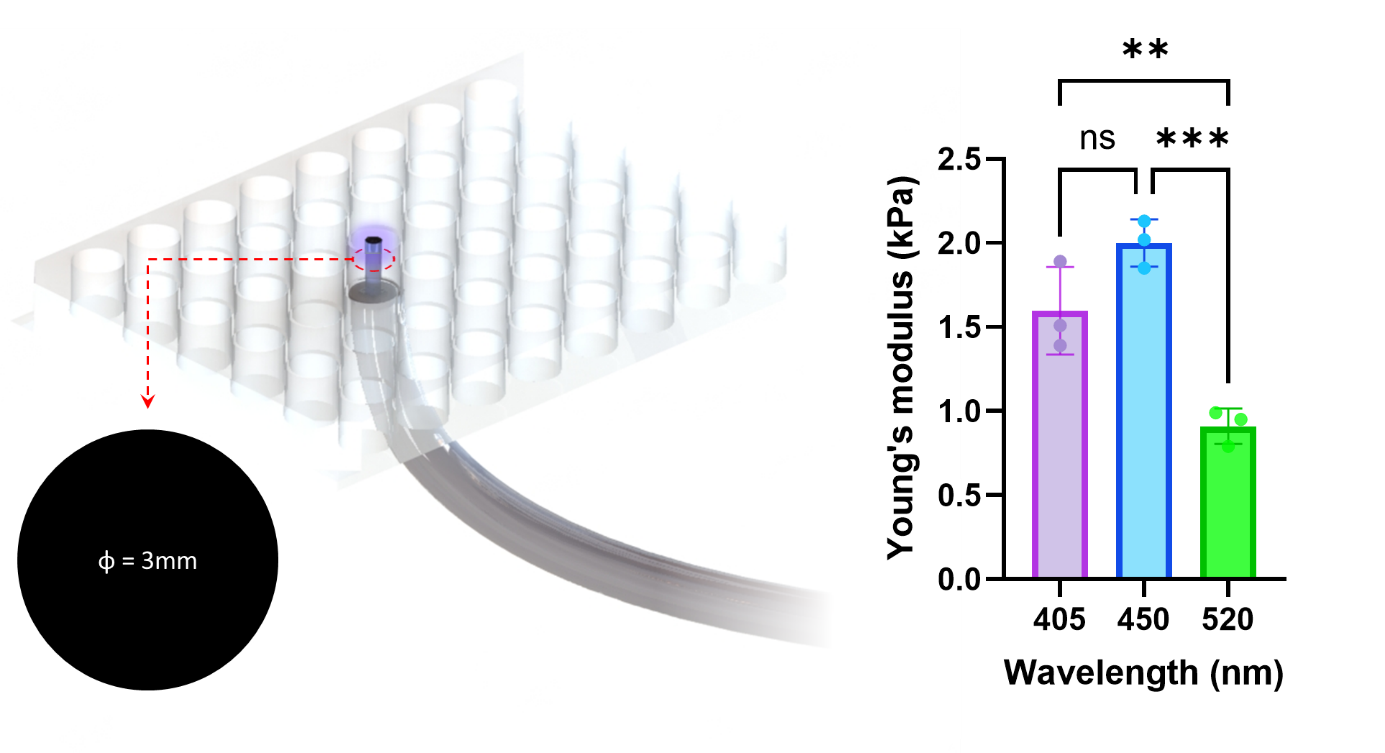


**Figure S4.** Setup for fabrication of cylindrical constructs for mechanical testing (left), where a circular image (diameter ɸ = 3 mm) was projected into well plates containing the photoresin formulations (200 µl/well). ** represent p<0.01, *** represent p<0.001.


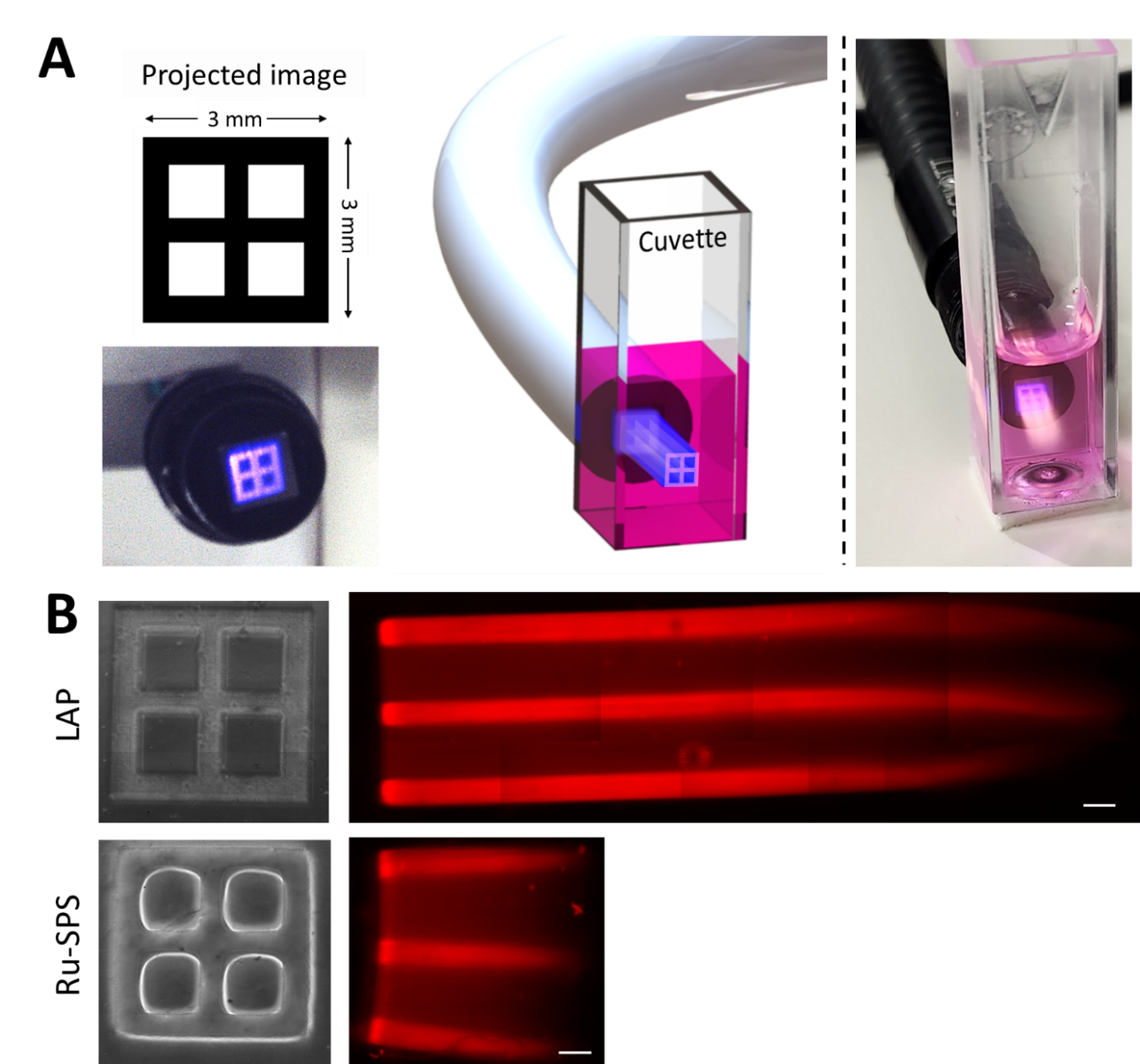


**Figure S5. A.** Projection of image patterns into 10 mm cuvettes. **B.** Sectional views of the crosslinked constructs, which demonstrate higher penetration depth in the LAP-containing photoresins in comparison to those containing Ru-SPS. Scale bar = 500 µm.


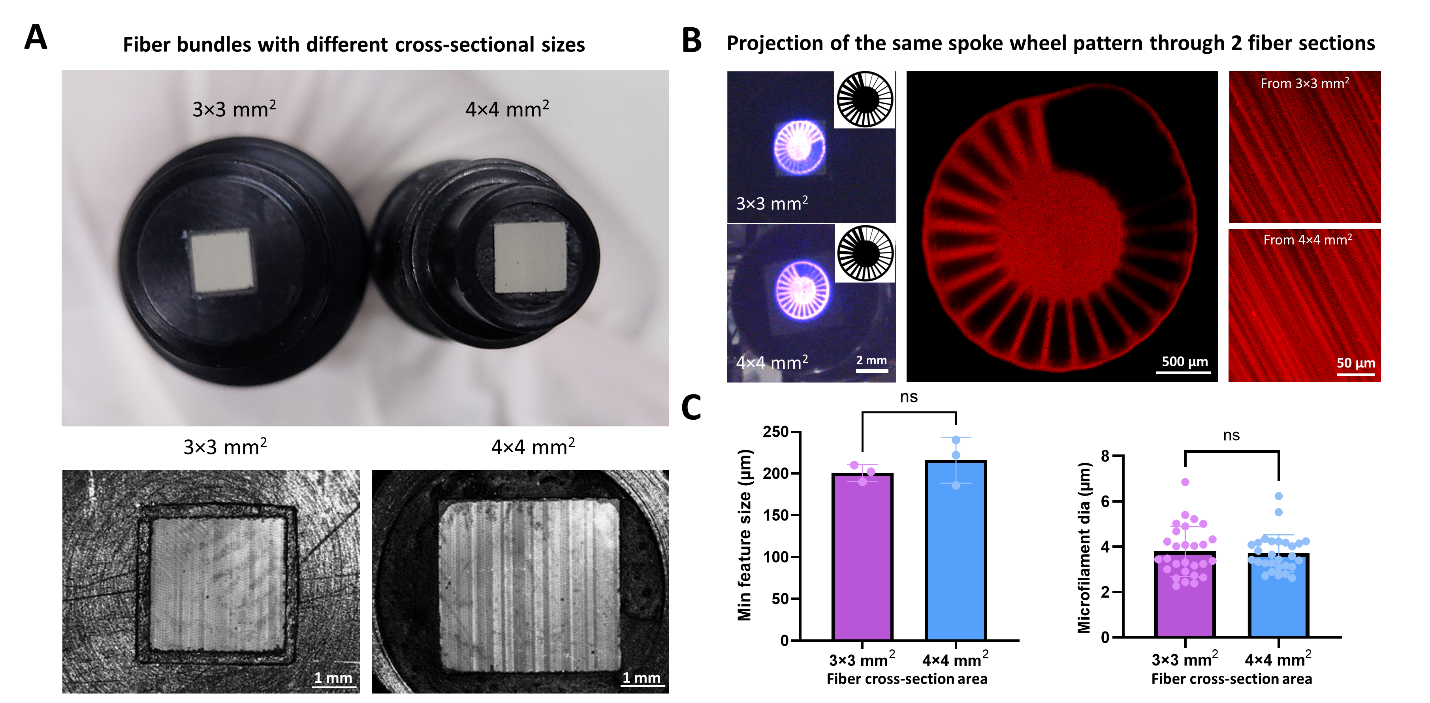


**Figure S6.** **A.** Comparison of different sizes of fiber bundles in the FaSt-Light apparatus, where two different fiber sections (3×3 mm^2^ and 4×4 mm^2^) were used, each of which constituted the same blocks of fiber arrays (each block is a 6×6 grid of individual fibers). Here, the number of blocks were larger in the 4×4 mm^2^ fiber bundle. **B.** Projected spoke wheel image (ɸ = 3 mm) from the two different fiber bundles and the crosslinked photoresin and its microfilament distribution. **C.** The corresponding results of minimum feature size and microfilament diameter.


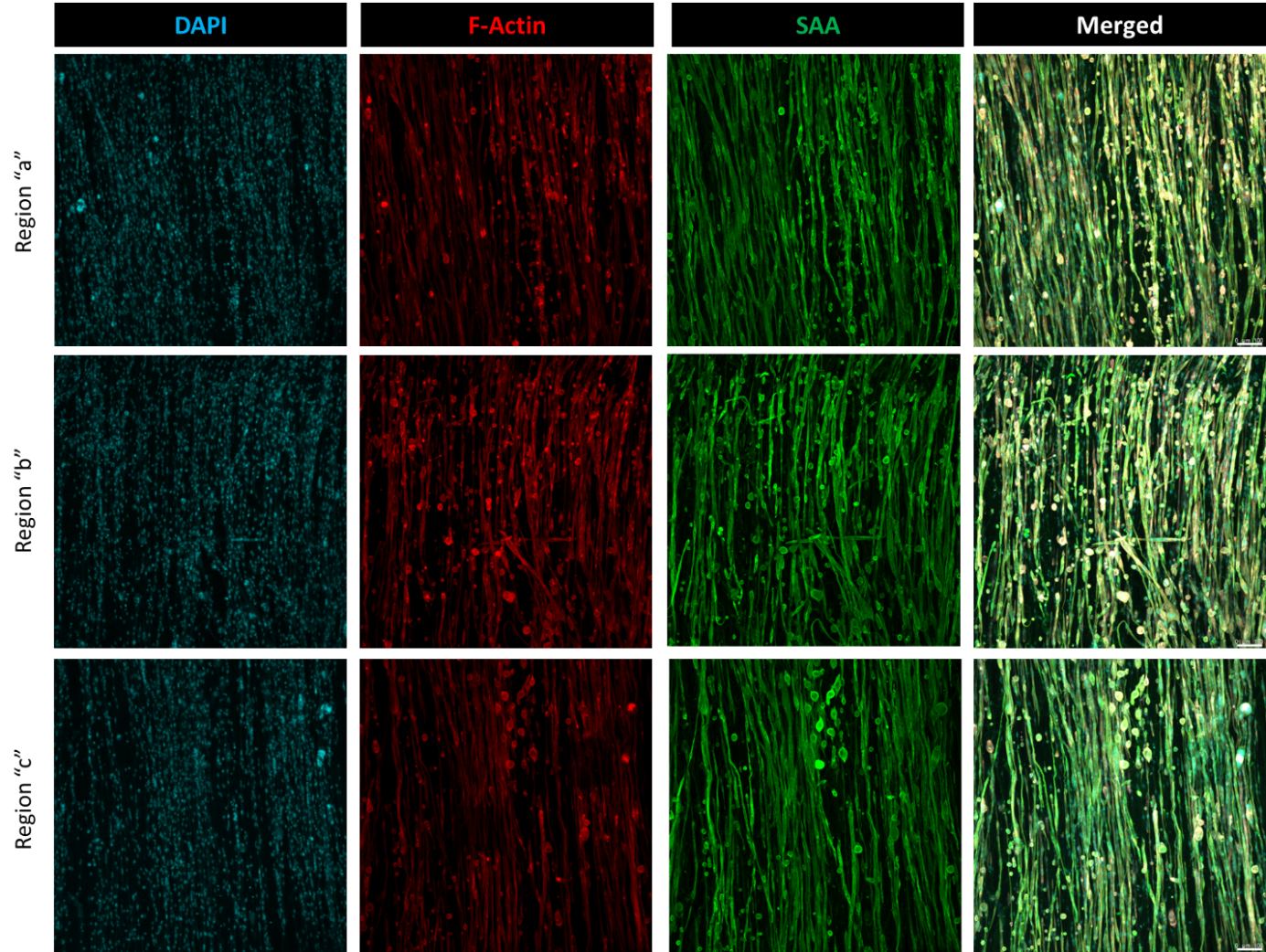


**Figure S7.** Micrographs of myotube distribution in the different regions of the muscle constructs biofabricated using the fiber bundle. All three regions along the constructs demonstrated similar density of myotubes. Scale bar (bottom right in the merged panel) = 100 µm.

**
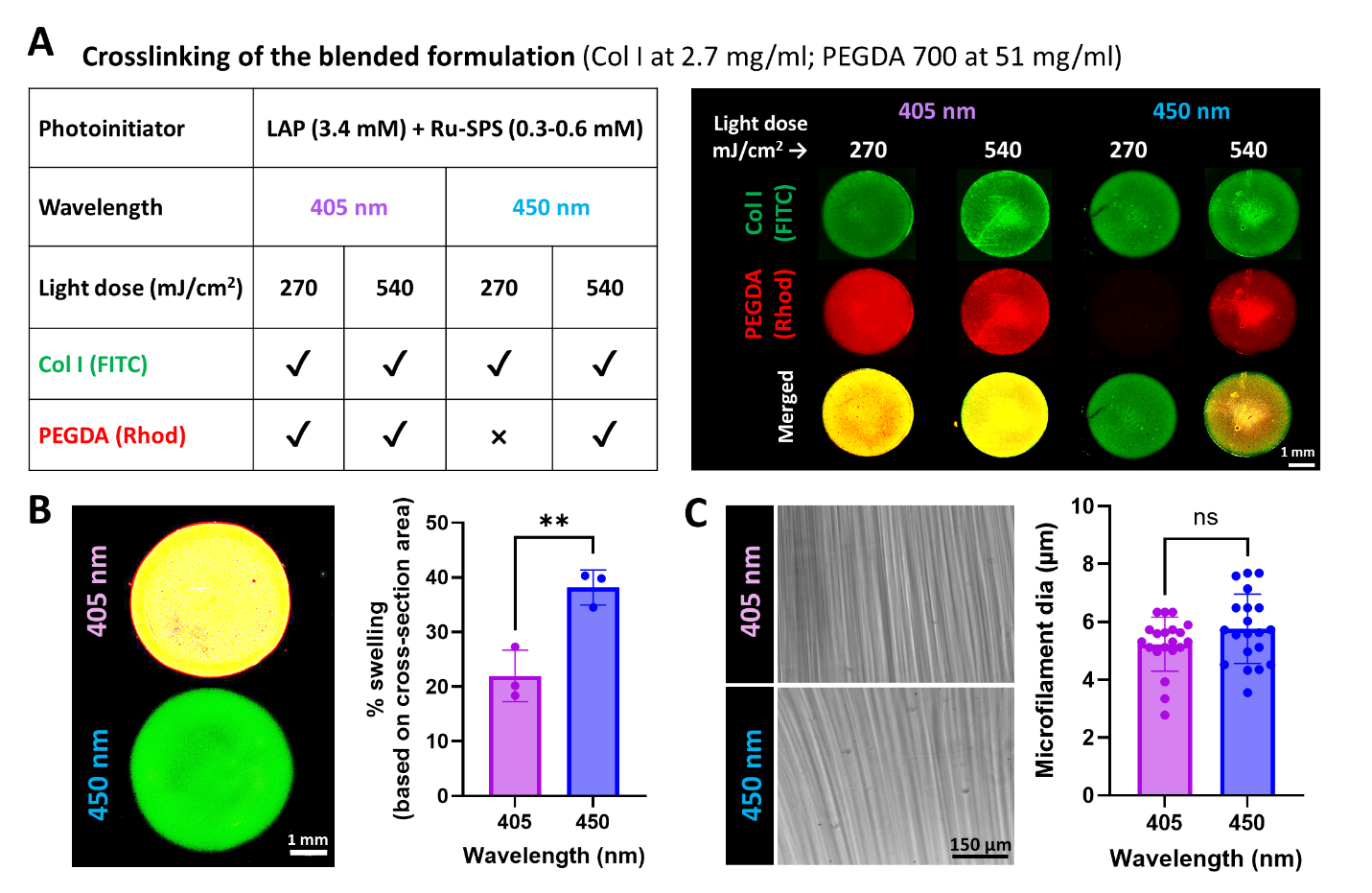
**

**Figure S8. A.** Light dose response of the blended resin formulation comprising of Collagen I at 2.7 mg/ml, PEGDA at 51 mg/ml, LAP at 3.4 mM and Ru-SPS at 0.3-0.6 mM, respectively, in DI water. Here, the PEGDA (Rhodamine labeled) demonstrated negligible crosslinking at 450 nm at lower light doses. **B.** The hydrogels swelled when kept at 37°C in mQ water for 4 hours, with higher swelling observed for the gels crosslinked at 450 nm, which is likely due to the negligible amount of PEGDA crosslinks in the network. A light dose of 270 mJ/cm^2^ was used for crosslinking at each wavelength. ** represents p < 0.01. **C.** The microfilament diameters of the constructs crosslinked at each wavelength were similar, albeit with higher average diameter in the formulations crosslinked at 450 nm which is likely due to increased swelling in the constructs.

**Figure S9.** Light transmission through skin tissue (0.64 ± 23 mm thickness) at different wavelengths in the FaSt-Light apparatus. *** represents p < 0.001 and **** represents p < 0.0001.


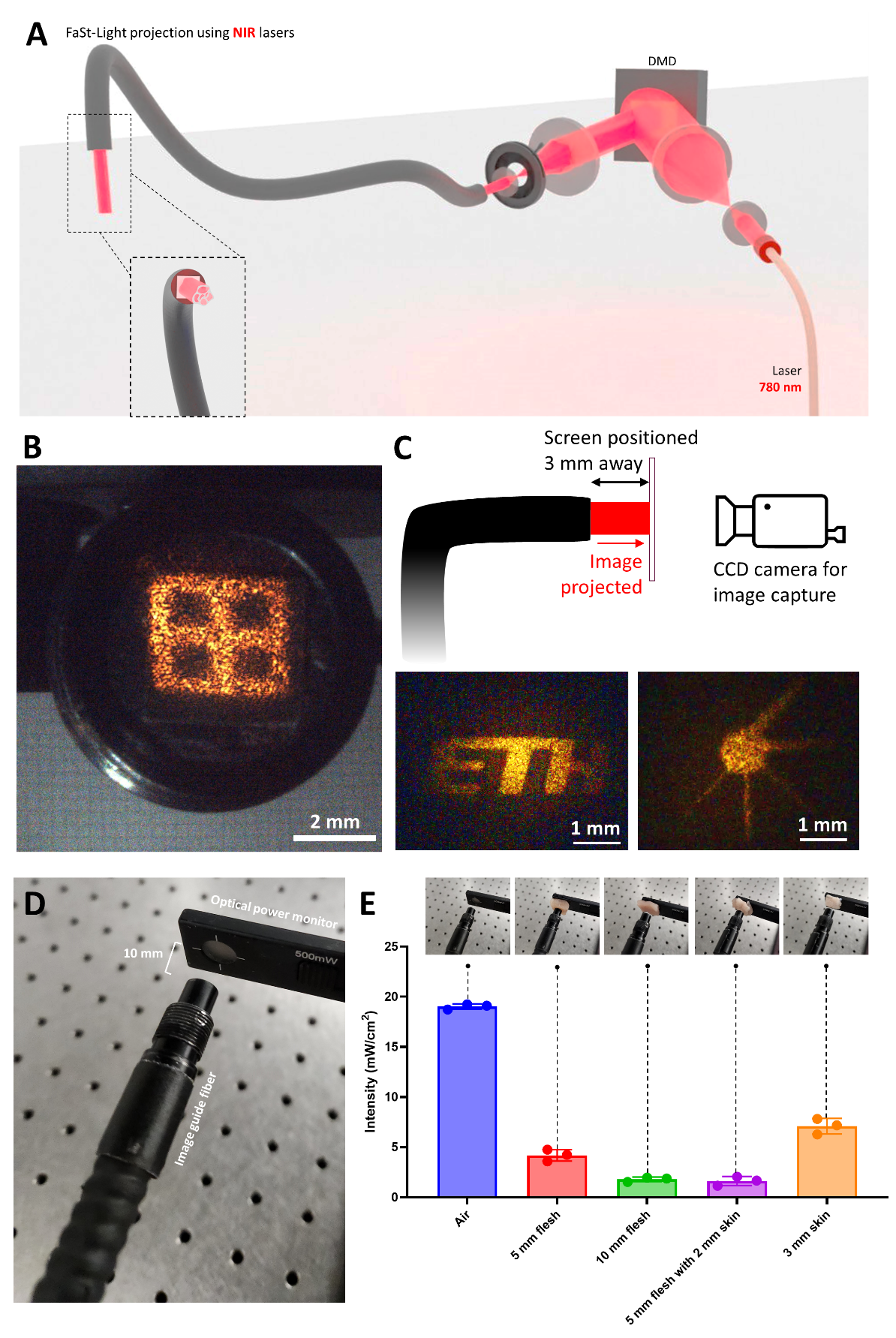


**Figure S10. Light projection at near infrared wavelength. A.** The multispectral laser module was decoupled and an NIR laser (780 nm) was coupled to the FaSt-Light apparatus. **B.** Projection of a window pattern directly from the fiber. **C.** Images of the ETH logo and the resolution test image projected from the fiber onto a white screen. **D.** Power measurements of red light from the fiber bundle with different sizes of tissues placed between the fiber and the power meter.
